# Matrix Rigidity Mechanoprimes Microglia for Inflammation Through Cytoskeletal‐to‐Nuclear Signaling and 3D Spatio‐Epigenomic Remodeling

**DOI:** 10.1002/advs.77736

**Published:** 2026-09-27

**Authors:** Yu Xuan Meng, Jaegeon Joo, Yu Meng Li, Cheng Ji Li, Ali Taghizadeh, Seung Jae Shin, Buuvee Bayarkhangai, Tae‐Woo Ha, Sak Lee, Jaewoo Chung, Bo‐Eun Yoon, Hye Sung Kim, Inkyung Jung, Jung‐Hwan Lee, Kam W. Leong, Hae‐Won Kim

**Affiliations:** ^1^ Institute of Tissue Regeneration Engineering (ITREN) Dankook University Cheonan Republic of Korea; ^2^ Institute of Toxicology, College of Preventive Medicine Third Military Medical University Chongqing China; ^3^ Department of Biological Sciences Korea Advanced Institute of Science and Technology (KAIST) Daejeon Republic of Korea; ^4^ Department of Nanobiomedical Science & BK21 NBM Global Research Center for Regenerative Medicine Dankook University Cheonan Republic of Korea; ^5^ Department of Molecular Biology Dankook University Cheonan Republic of Korea; ^6^ Department of Oral Pathology School of Dentistry Seoul National University Seoul Republic of Korea; ^7^ Department of Neurosurgery Dankook University Cheonan Republic of Korea; ^8^ Mechanobiology Dental Medicine Research Center Dankook University Cheonan Republic of Korea; ^9^ Cell & Matter Institute Dankook University Cheonan Republic of Korea; ^10^ Department of Biomaterials Science College of Dentistry Dankook University Cheonan Republic of Korea; ^11^ Department of Biomedical Engineering Columbia University New York New York USA

**Keywords:** chromatin accessibility, matrix rigidity, mechanoCREs, microglia, neuroinflammation

## Abstract

Microglia play a pivotal role in modulating the pathophysiology of the central nervous system, including disease progression and injury repair. While the biochemical factors governing microglial activation are well understood, the impact of biophysical cues, such as extracellular matrix (ECM) mechanics, remains largely unexplored. Here, we demonstrate how matrix rigidity mechanoprimes microglia for inflammation through coordinated cytoskeletal‐to‐nuclear signaling and 3D spatio‐epigenomic remodeling. In response to rigid matrices, microglia undergo progressive actin cytoskeletal remodeling, morphological adaptation, and nuclear deformation. ATAC‐seq reveals that rigidity redistributes rather than globally increases chromatin accessibility: most differentially accessible regions lose accessibility, whereas a defined subset gains focal accessibility at inflammation‐associated loci, establishing a permissive state for subsequent activation. Upon LPS challenge, rigidity amplifies inflammatory responses through NF‐κB activation and cytoskeleton‐dependent MRTF‐A nuclear translocation. Integrating ChIP‐seq and Hi‐C identifies rigidity‐responsive *cis*‐regulatory elements (mechanoCREs) and shows that rigidity promotes long‐range chromatin interactions linking distal mechanoCREs to inflammatory genes. Together, these findings establish ECM rigidity as a key regulator of microglial inflammation by coupling actin‐mediated mechanotransduction to chromatin accessibility and higher‐order genome organization, providing a mechanistic basis for mechanically amplified neuroinflammation.

## Graphical abstract

Pathological tissue rigidity mechanoprimes microglia by enhancing actin cytoskeleton–nucleus coupling and chromatin opening at rigidity‐responsive cis‐regulatory elements (mechanoCREs). Subsequent NF‐κB/p65 signaling converges on this permissive regulatory state to amplify inflammatory gene expression and microglial activation.

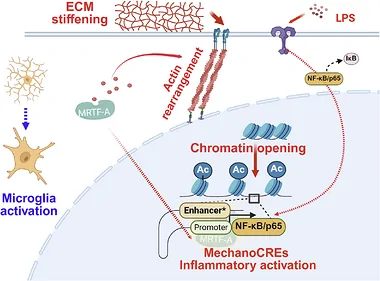

## 1 Introduction

Microglia play a central role in regulating central nervous system (CNS) homeostasis and pathophysiology. Through dynamic transitions among functional states, they mediate immune surveillance and coordinate responses to injury and infection [1]. This functional plasticity is shaped by diverse microenvironmental factors, including soluble signaling molecules (e.g., cytokines, chemokines), pathogen‐associated molecular patterns, and damage‐associated molecular patterns [2, 3, 4]. Microglia exist along a continuum of functional states rather than as a simple binary switch. In the healthy CNS, homeostatic microglia extend highly ramified processes that continuously survey the parenchyma, prune synapses, clear apoptotic debris, and provide trophic support to neurons—functions supported by distinct transcriptional and epigenetic programs. Upon injury, infection, or degeneration, microglia retract their processes, adopt less ramified, often amoeboid morphologies, and shift toward reactive states characterized by cytokine secretion, altered phagocytic activity, and activation of inflammatory transcriptional programs, including NF‐κB signaling. Because these state transitions require coordinated remodeling of both cellular morphology and gene regulatory programs, microglia are well suited to sense and integrate mechanical cues within their microenvironment.

Along with these soluble biochemical signals, biophysical cues, particularly tissue mechanics, including extracellular matrix (ECM) rigidity, have emerged as key regulators of microglial activation [5, 6]. Notably, altered tissue mechanics have been reported across multiple CNS disorders associated with neuroinflammation [7, 8, 9, 10, 11, 12, 13]. Reported stiffness values vary considerably with measurement modality, spatial scale, and disease context. Healthy brain parenchyma is generally soft, with reported values commonly falling within the ∼1–10 kPa range [14, 15], whereas inflammatory and fibrotic remodeling can produce pronounced, spatially localized changes in tissue stiffness. Traumatic brain injury, for instance, can be accompanied by local tissue stiffening associated with reactive gliosis and glial scar formation, processes implicated in secondary injury and neurological dysfunction [16, 17]. Altered neural tissue mechanics have also been reported in neurodegenerative diseases, including Alzheimer's disease, Parkinson's disease, and amyotrophic lateral sclerosis [18, 19]. Marked focal stiffening has additionally been observed in brain tumors, particularly in experimental glioblastoma models: bulk compressive measurements of U87 glioblastoma xenografts have reached ∼26.7 kPa [20], although in vivo elastography of patient tumors can yield values comparable to surrounding parenchyma, highlighting differences among measurement modalities and models, as well as substantial intratumoral heterogeneity.

Rather than defining a single pathological stiffness, these reports collectively delineate a heterogeneous and context‐dependent mechanical spectrum across which microglia need to operate. Accordingly, changes in neural tissue mechanics may critically shape microglial state transitions and inflammatory responses. Elucidating how microglia sense these mechanical alterations and integrate them with biochemical inflammatory cues could thus reveal previously unrecognized disease mechanisms and potential therapeutic targets for neuroinflammatory conditions; however, the underlying mechanotransductive mechanisms remain poorly understood.

Recent findings highlight that peripheral immune cells, such as macrophages and dendritic cells, sense ECM rigidity and viscoelasticity through cytoskeletal mechanotransduction, which in turn drives morphological adaptation and gene expression changes [21, 22, 23]. Crucially, these mechanotransduction events extend into the nucleus, altering its physical structure and remodeling its chromatin architecture [24, 25, 26]. However, in microglia, the interplay between matrix mechanics, chromatin remodeling, and inflammatory gene regulation remains largely underexplored. Elucidating these mechanobiological links is critical for deciphering how pathological tissue stiffening drives neuroinflammatory mechanisms, ultimately revealing novel therapeutic targets for various CNS disorders.

Here, we investigate how matrix rigidity mechanoprimes microglia for inflammatory responses, with a particular focus on its interplay with biochemical stimuli. We used 1 and 23 kPa substrates to span the compliant and stiff extremes of the reported range described above, rather than to replicate any single pathological tissue value. We demonstrate that microglia actively mechanosense and adapt to matrix rigidity through progressive actin cytoskeletal remodeling and nuclear deformation. Notably, these rigidity‐driven mechanosensitive events are significantly amplified in the presence of inflammatory biochemical stimuli. In particular, multi‐omics profiling, integrating ATAC‐seq and ChIP‐seq, reveals that matrix rigidity induces nuclear chromatin remodeling that redistributes chromatin accessibility, reducing it at loci linked to homeostatic neural‐interaction programs while focally increasing it at inflammation‐associated regulatory elements. Intriguingly, the majority of these chromatin alterations occur at distal regions, suggesting the existence of mechanosensitive *cis‐*regulatory elements (mechanoCREs). High‐throughput chromosome conformation capture (Hi‐C) analysis further confirms that under rigid matrix conditions, these mechanoCREs establish de novo long‐range chromatin interactions with upregulated inflammatory genes. Together, this study establishes tissue rigidity as a fundamental regulator of microglial inflammation through cytoskeletal‐to‐nuclear mechanotransduction and 3D spatio‐epigenomic remodeling, underscoring the critical role of ECM mechanics in regulating microglia‐associated neuroinflammatory responses.

## 2 Results and Discussion

### 2.1 Microglia Mechanosense Matrix Rigidity via Progressive Actin Cytoskeletal Remodeling and Nuclear Deformation

**FIGURE 1:**
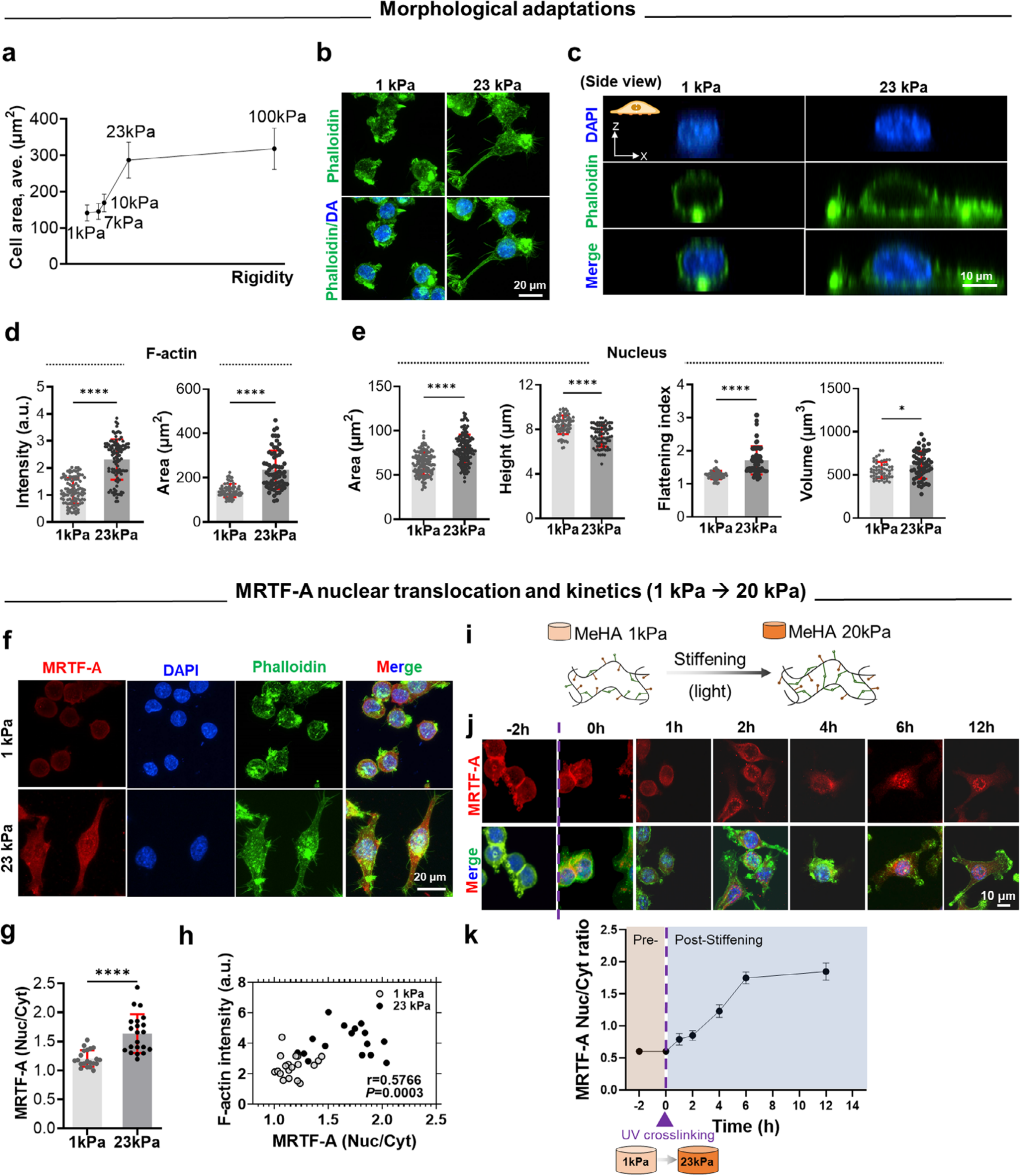
Microglia adapt morphology, nuclear architecture, and MRTF‐A nuclear translocation in response to matrix rigidity. (a) Quantitative analysis of microglial (BV2) cell area on poly (acrylamide) (PAA) gels of varying stiffness (1, 7, 10, 23, and 100 kPa) coated with 0.05 mg/mL fibronectin. (b,c) Representative confocal micrographs of microglia cultured on compliant (1 kPa) and rigid (23 kPa) matrices for 12 h. Top–down (*XY*) and orthogonal (*XZ*) views show F‐actin (green) and nuclei (DAPI, blue). Cells exhibit a rounded morphology on 1 kPa compared to an enlarged, spindle‐shaped morphology with robust cytoskeletal processes on 23 kPa. (d) Quantification of F‐actin intensity and 2D projection area. (e) Characterization of nuclear mechanoadaptation, including 2D projection area, height, flattening index, and 3D volume. (f,g) Representative confocal images and quantification of the nuclear‐to‐cytoplasmic (Nuc/Cyt) ratio of MRTF‐A, showing increased nuclear translocation on stiffer substrates. (h) Pearson's correlation analysis between MRTF‐A nuclear localization and F‐actin intensity (*p* = 0.0003). (i–k) Kinetics of MRTF‐A nuclear translocation on dynamically stiffening substrates: (i) Schematic of the methacrylated hyaluronic acid (MeHA) hydrogel system, which stiffens from 1 to 20 kPa upon 30 s of UV exposure, and (j,k) immunofluorescence imaging and temporal quantification show MRTF‐A nuclear translocation over 12 h following matrix stiffening. The violet dotted line indicates the time point of UV crosslinking. Cells cultured on 1 kPa hydrogels were stiffened to 20 kPa by UV exposure at *t* = 0 (arrowhead). Time points span from −2 h (before stiffening) to 12 h, with UV‐induced stiffening applied at *t* = 0 (arrowhead). The −2 h time point represents the pre‐stiffening baseline, and subsequent time points represent the adaptation phase after stiffening. Data are presented as mean ± SD from three independent biological experiments. *n*‐values represent the number of cells/ROIs analyzed per condition as specified in the panels. Statistical significance was determined via Student's *t*‐test: ****p < 0.0001, ***p < 0.001, **p < 0.01, *p < 0.05; ns, not significant (*p* > 0.05).

To investigate whether microglia mechanosense matrix rigidity, we cultured microglial (BV2) cells on fibronectin‐coated polyacrylamide (PAA) hydrogels of varying stiffness (1, 7, 10, 23, and 100 kPa). Optical microscopy revealed a profound, rigidity‐dependent increase in cell area. This spreading behavior increased substantially up to 23 kPa and reached a near‐plateau at 100 kPa (Figure 1a; Figure S1a) [27], demonstrating that microglia undergo morphological adaptation in response to the underlying matrix mechanics.

Based on these observations and established rigidity values for healthy and stiffened brain tissues [7, 28], we selected 1 and 23 kPa hydrogels to represent compliant and rigid gel matrices, respectively, recapitulating the mechanics of normal and pathological brain environments. Immunostaining of cells revealed that F‐actin cytoskeletal architecture differed substantially between compliant and rigid matrices, as observed in z‐stacked top–down (*XY*) and orthogonal (*XZ*) views, although both conditions exhibited similarly pronounced actin ruffling along the apical plane (Figure 1b,c; Figure S1b,c). Cells on rigid matrices developed robust actin cytoskeletons with an enlarged, spindle‐shaped morphology [29], consistent with typical BV2 microglial mechanoadaptation to rigid substrates [27]. In contrast, cells on compliant matrices displayed limited cytoskeletal processes and maintained a rounded, contracted shape. Quantitative analysis of F‐actin intensity and 2D projected cell area confirmed significantly higher values on 23 kPa compared to 1 kPa (Figure 1d).

Of note, orthogonal (*XZ*) cross‐sectional imaging revealed distinct nuclear deformation in cells cultured on 23 kPa, contrasting with the spherical nuclei observed on 1 kPa (Figure 1c). To further characterize this nuclear mechanoadaptation, we quantified 2D projection area, height, flattening index, and 3D volume (nucleus indices illustrated in Figure S2) [30]. Cells on rigid matrices exhibited a significantly expanded 2D nuclear area coupled with a decreased nuclear height, resulting in a markedly elevated nuclear flattening index. While nuclear volume changes were relatively minimal, a slight increase was observed on rigid matrices (Figure 1e). Consistently, primary microglia isolated from neonatal mice displayed comparable rigidity‐driven adaptations, including enhanced cell spreading, F‐actin organization, and increased nuclear flattening on rigid substrates (Figure S3).

Importantly, this observed increase in nuclear flattening driven by an expanded nuclear area at the expense of decreased nuclear height demonstrated a strong positive correlation with changes in whole cell area and F‐actin intensity. This correlation suggests that the progressive development of the actin cytoskeleton in response to elevated matrix rigidity acts as the primary mechanical driver of nuclear deformation.

Given the substantial development of F‐actin on rigid matrices, we hypothesized that the key mechanosensor, myocardin‐related transcription factor A (MRTF‐A), undergoes nuclear translocation following its dissociation from the G‐actin pool during extensive F‐actin polymerization [21]. Indeed, rigid matrices significantly increased the nuclear translocation of MRTF‐A (Figure 1f,g), a phenomenon that positively correlated with F‐actin intensity (Figure 1h). This enhanced nuclear transport is also linked to the physical opening of nuclear pores, mechanically induced by cell and nuclear flattening [31]. Transmission electron microscopy analysis revealed a significant enlargement (∼20%) in nuclear pore size at 23 kPa compared to 1 kPa (90.5 nm vs. 75.4 nm, Figure S4a,b). Moreover, real‐time fluorescence imaging demonstrated that a 70 kDa dextran, utilized as a model macromolecule, translocated into the nucleus more efficiently on rigid matrices (Figure S4c). Consequently, the enhanced nuclear accumulation of MRTF‐A is attributed synergistically to F‐actin polymerization and the concurrent increase in nuclear pore size driven by mechanoadaptive flattening.

We further evaluated the kinetics of MRTF‐A nuclear translocation over culture time. The temporal increase in MRTF‐A levels upon matrix stiffening was monitored using a stiffening methacrylated hyaluronic acid (MeHA) hydrogel platform [32] (Figure S5). Cells were cultured on compliant MeHA gels (1 kPa) for 12 h, after which the matrix was stiffened to a rigid state (20 kPa) via UV light exposure for 30 s (Figure 1i). MRTF‐A nuclear translocation was then monitored over the subsequent 12 h. Immunofluorescence imaging (Figure 1j) demonstrated that MRTF‐A nuclear translocation, which was initially minimal on the compliant gel (ratio of ∼0.6), gradually increased following matrix stiffening over a 6 h period (reaching a ratio of ∼1.7), and maintained a near‐plateau through 12 h (Figure 1k). Together, these results demonstrate the capacity of microglial cells to mechanosense the alterations in underlying matrix rigidity. This mechanosensing dictates the progressive remodeling of actin cytoskeletal architecture and coordinated morphological adaptations, including nuclear deformation—mechanoadaptive events that facilitate the nuclear translocation of the transcriptional coactivator MRTF‐A.

### 2.2 Matrix Rigidity Remodels Chromatin Architecture, Increasing Gene Accessibility for Microglial Activation

**FIGURE 2:**
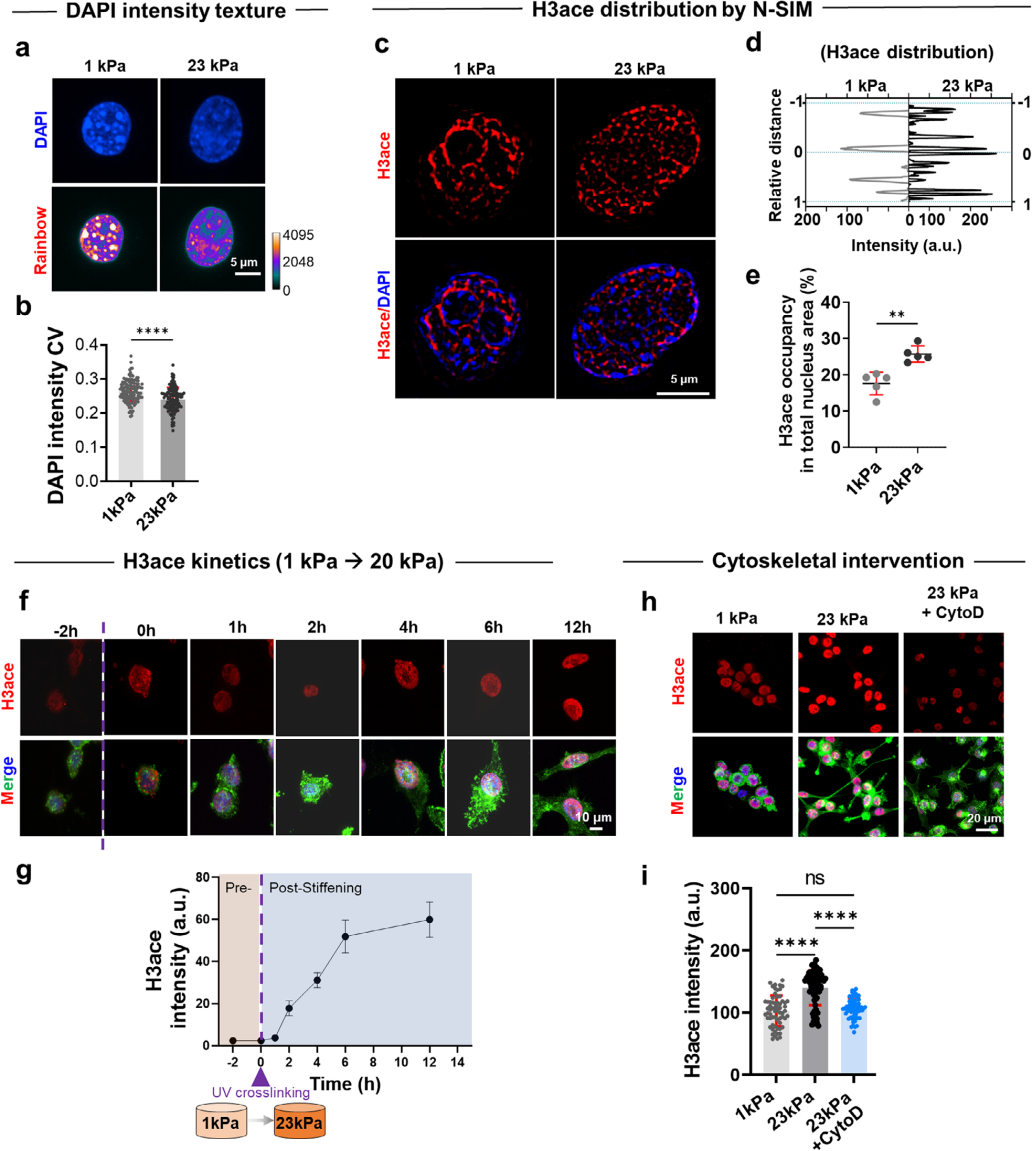
Matrix rigidity drives microglial nuclear chromatin modifications, mediated by actin cytoskeletal development. (a,b) Confocal imaging of microglial (BV2) cells cultured on compliant (1 kPa) and rigid (23 kPa) matrices for 12 h. Images are maximum‐intensity projections of confocal z‐stacks: (a) Representative images of nuclear DNA stained with DAPI (blue) alongside a rainbow intensity map, and (b) quantification of nuclear condensation, defined as the coefficient of variation (standard deviation/mean intensity of DAPI staining) (*n* = 132). (c–e) Super‐resolution N‐SIM analysis of nuclear architecture: (c) Representative images of nuclei stained for DNA (DAPI, blue) and histone H3 acetylation (H3ace, red), detailing nanoscale chromatin patterns. Images are single optical sections acquired at the nuclear mid‐plane by N‐SIM super‐resolution microscopy. (d) Normalized fluorescence intensity profile of H3ace distributed along the indicated vector (white arrow). (e) Semi‐quantification of H3ace occupancy relative to total nuclear area (*n* = 5). (f,g) Temporal monitoring of H3ace levels over 12 h using a dynamically stiffening MeHA gel platform (transitioning from 1 to 20 kPa). The violet dotted line indicates the time point of UV crosslinking. Stiffening was induced by UV exposure at *t *= 0 (arrowhead). Time points span from −2 h (before stiffening) to 12 h, with UV‐induced stiffening applied at *t *= 0 (arrowhead). The −2 h time point represents the pre‐stiffening baseline, and subsequent time points represent the adaptation phase after stiffening. (f) Immunofluorescence images of H3ace co‐stained with F‐actin, and (g) corresponding semi‐quantification of H3ace intensity (*n* = 20). (h,i) Impact of cytoskeletal tension disruption via cytochalasin D (CytoD) treatment on H3ace levels: (h) Confocal images of H3ace (red), F‐actin (green), and nuclei (blue) following 11 h of culture and a subsequent 1 h CytoD treatment, and (i) semi‐quantification of average H3ace intensity across experimental conditions (*n* = 64).

Given the significant nuclear deformation induced by matrix rigidity, we sought to explore its impact on microglial chromatin reorganization. DAPI intensity texture analysis in both BV2 cells and primary microglia revealed a significant decrease in the coefficient of variation on rigid matrices, consistent with reduced chromatin compaction (Figure 2a,b; Figure S6a,b). We note that DAPI texture provides an indirect proxy for chromatin compaction and is sensitive to nuclear area, height, and imaging geometry; accordingly, we corroborated these observations with direct molecular readouts (H3ace immunostaining, ATAC‐seq, and ChIP‐seq). Subsequent super‐resolution N‐SIM imaging, following immunostaining for acetylated histone H3 (H3ace), demonstrated a more evenly distributed and densely occupied H3ace signal within the nucleus on rigid matrices, indicating mechanically driven chromatin decondensation (Figure 2c–e; Figure S7). Primary microglia displayed similar stiffness‐dependent increases in H3ace, supporting conserved mechanosensitive chromatin remodeling across microglial models (Figure S6c,d). These findings imply that mechanical cues, specifically matrix rigidity, prime nuclear chromatin in the absence of exogenous inflammatory biochemical signals [33, 34]. The kinetics of this H3ace increase over culture time were further examined using a dynamically stiffening MeHA gel platform (Figure 2f,g). Intriguingly, these time‐dependent changes closely mirrored those observed for MRTF‐A nuclear translocation (as detailed previously in Figure 1j,k). Next, to confirm the dependency on the F‐actin cytoskeleton, we performed pharmacological inhibition with cytochalasin D (CytoD). This cytoskeletal disruption reversed the elevated H3ace levels induced by rigid matrices, restoring them nearly to the baseline levels observed on compliant matrices (Figure 2h,i).

**FIGURE 3:**
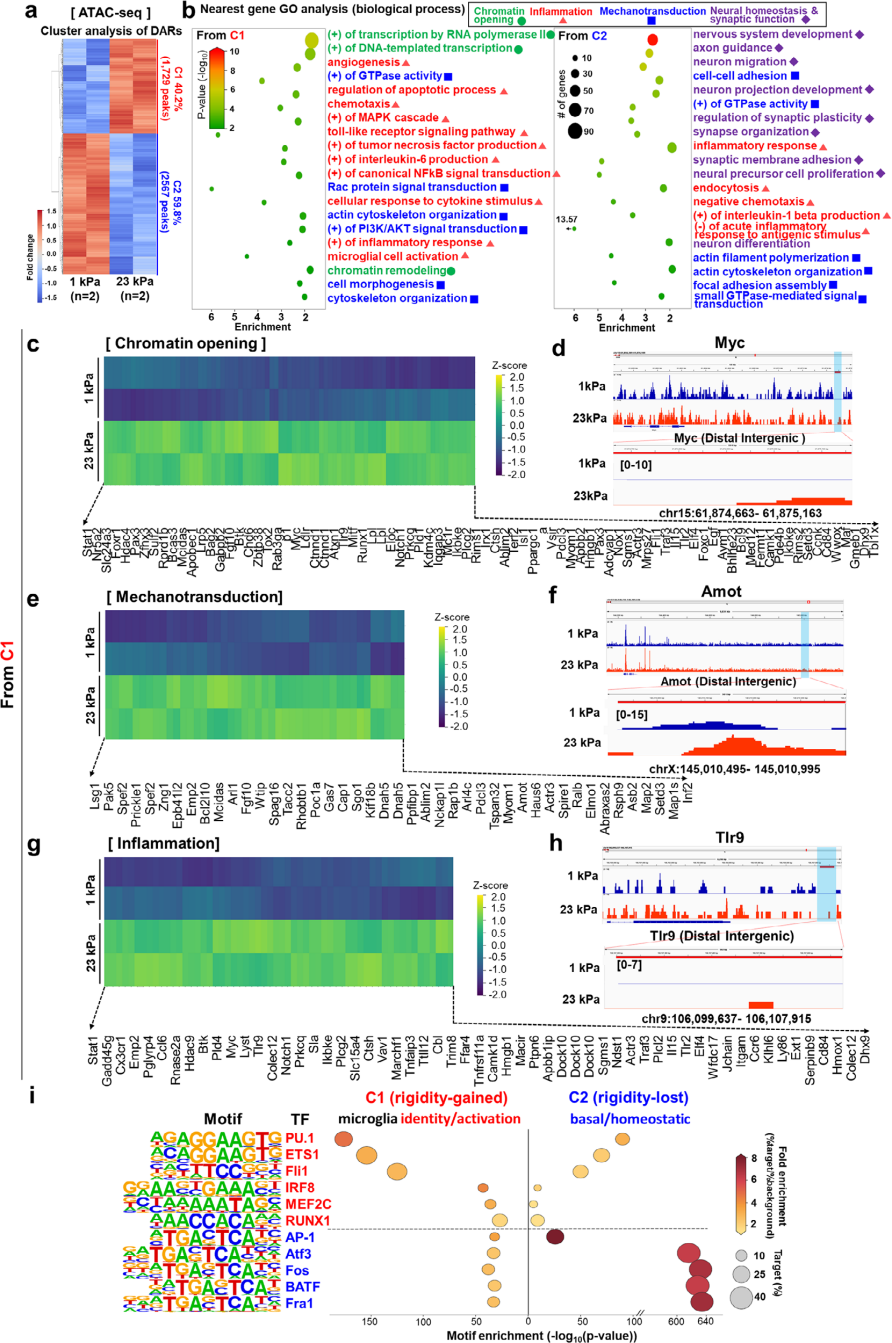
Matrix rigidity redistributes chromatin accessibility, focally increasing it at loci associated with chromatin opening, mechanotransduction, and inflammation. ATAC‐seq analysis of differentially accessible regions (DARs) in microglial cells cultured on compliant and rigid matrices (*n* = 2 biological replicates). (a,b) Global DAR analysis (fold change ≥ 1.3, *p* < 0.05). (a) Cluster analysis of all 4296 DARs identified between 1 and 23 kPa. DARs segregated into two clusters: C1, comprising 1729 regions (40.2%) with increased accessibility on rigid matrices, and C2, comprising 2567 regions (59.8%) with decreased accessibility. Color denotes fold change. (b) Gene Ontology (biological process) enrichment of nearest genes for C1 (left) and C2 (right), analyzed under identical parameters and background. Bubble size denotes gene count, and color denotes *p*‐value. Terms with *p* < 0.01 are shown. Term categories are color‐coded as indicated. In all GO term labels, (+) and (−) denote positive and negative regulation, respectively. (c–h) Representative loci from C1, selected under a more stringent threshold (fold change ≥ 1.5, *p* < 0.05) to highlight the most robustly affected regions. (c) Heatmap displaying increased promoter accessibility on rigid matrices for genes associated with positive regulation of gene expression (GO: 0010628) and RNA polymerase II transcription (GO: 0045944). (d) Representative ATAC‐seq signal tracks for the *Myc* locus. (e) Heatmap showing enhanced promoter accessibility for mechanotransduction‐related genes, encompassing myosin light chain kinase activity (GO: 0004687), GTPase activity (GO: 0003924), and Rho protein signal transduction (GO: 0007266). (f) ATAC‐seq signal tracks for the representative cytoskeletal regulatory gene *Amot*. (g) Heatmap illustrating increased promoter accessibility of inflammation‐related genes enriched in immune system processes (GO: 0006955) and inflammatory responses (GO: 0006954). (h) ATAC‐seq signal tracks for the representative inflammatory gene *Tlr9*. For all ATAC‐seq tracks (d,f,h), the *y‐*axis represents peak signal intensity, the *x‐*axis indicates genomic position, and non‐coding DARs are magnified. Additional representative tracks are provided in Figure S13. (i) HOMER motif enrichment analysis performed separately for the C1 and C2 clusters, each against a random genomic background. For each transcription factor, the motif logo (left) and enrichment in C1 (rigidity‐gained; left of the axis) and C2 (rigidity‐lost; right of the axis) are shown. Bubble size denotes the percentage of target sequences containing the motif, color denotes fold enrichment (%target/%background), and the *x*‐axis shows motif enrichment as −log_10_ (*p*‐value); the C2 axis is broken to accommodate the very high AP‐1 enrichment values. C1 was dominated by microglia identity/activation regulators (PU.1, ETS1, Fli1, IRF8, MEF2C, and RUNX1), whereas C2 was dominated by AP‐1 family factors (Fos, Jun, Fra1, BATF, and Atf3) marking basal/homeostatic enhancers. The complete de novo motif output for both clusters is provided in Figure S14.

To further investigate the chromatin accessibility of genes related to matrix‐induced chromatin reorganization, we performed an assay for transposase‐accessible chromatin with high‐throughput sequencing (ATAC‐seq). Experiments were conducted in the absence of exogenous biochemical stimuli after confirming the absence of mycoplasma contamination (Figure S8a). Furthermore, multiple quality control metrics for the ATAC‐seq data from both compliant and rigid matrices validated the high quality of the sequencing results (Figure S8b–d). Cluster analysis of differentially accessible regions (DARs) demonstrated tight replicate clustering, with distinct accessibility patterns across identified genomic regions, as shown in the ATAC‐seq heatmap (Figure 3a). We identified 4296 DARs between compliant and rigid matrices. Notably, the majority of DARs became less accessible on rigid matrices: 2567 regions (59.8%, C2) showed reduced accessibility on 23 kPa, whereas 1729 regions (40.2%, C1) showed increased accessibility. Matrix rigidity therefore does not globally increase chromatin accessibility. Instead, it redistributes accessibility bidirectionally, and we examined both clusters to determine what distinguishes them.

To determine whether the two clusters differ in regulatory character, we compared their genomic distributions. C1 regions were significantly enriched at promoters relative to C2 (24.6% vs. 15%), whereas C2 regions were disproportionately located in gene‐poor distal intergenic space (43.2% vs. 33.3%) (Figure S9). Thus, although C2 contains more regions, C1 is markedly more concentrated at promoter‐proximal regulatory elements.

Gene Ontology analysis of nearest genes reinforced this distinction (Figure 3b). C1 regions were enriched for transcriptional activation and chromatin remodeling, for mechanotransduction processes including GTPase activity and actin cytoskeleton organization, and for inflammatory signaling spanning the full axis from receptor to effector—toll‐like receptor signaling, canonical NF‐κB signal transduction, MAPK cascade, and positive regulation of TNF and IL‐6 production. Notably, microglial cell activation itself was enriched among C1 genes, directly linking rigidity‐gained accessibility to the activation phenotype.

C2 regions were instead dominated by neural developmental and synaptic programs, including nervous system development, axon guidance, neuron migration, regulation of synaptic plasticity, and synapse organization—processes corresponding to the homeostatic functions of microglia in CNS surveillance, synaptic pruning, and neuronal interaction. Matrix rigidity therefore does not simply open or close chromatin; it repartitions accessibility between two functionally opposed programs, closing loci that support homeostatic neural interaction while opening loci that drive inflammatory activation.

Consistent with this reciprocal switch, inflammation‐related terms appeared in both clusters but with distinct membership: C1 was enriched for the canonical pro‐inflammatory activation cascade, whereas C2 contained terms associated with restraint of inflammation, including negative chemotaxis and negative regulation of the acute inflammatory response. Rigidity thus rewires, rather than uniformly amplifies, the inflammatory regulatory landscape. This bidirectional pattern is fully compatible with the global increase in H3 acetylation observed by imaging. Elevated H3 acetylation promotes broad, low‐amplitude chromatin decompaction, whereas ATAC‐seq peak calling preferentially captures focal, high‐contrast accessibility at discrete regulatory elements. The two assays therefore report on different scales of chromatin organization, and similar uncoupling between global acetylation states and discrete ATAC‐seq peaks has been reported in other contexts [35, 36, 37]. Indeed, functional enrichment via gene ontology (GO) analysis of highly accessible genes from nearest or *cis*‐regulatory regions [38, 39] revealed significant enrichment in key biological processes, including chromatin opening, mechanotransduction, and inflammation (Figure S10). These findings were further validated through molecular function term and Kyoto Encyclopedia of Genes and Genomes (KEGG) pathway analyses (Figure S11).

Notably, rigidity‐induced upregulated DARs corresponded to increased accessibility at loci associated with chromatin opening (Figure 3c), mechanotransduction (Figure 3e), and inflammation (Figure 3g). Further analysis of DARs identified significant enrichment (over 94%) in non‐exonic regions, particularly distal intergenic elements, suggesting that extensive non‐coding regulatory mechanisms underlie mechanosensitive microglial activation (Figure S12). Indeed, genome browser visualization highlighted specific non‐coding peak tracks for several important regulatory genes: the chromatin accessibility regulators (*Myc*, *Kdm4c*, *Chd6*, *Hmgb1*) [40, 41, 42, 43] (Figure 3d; Figure S13), cytoskeletal regulators (*Amot, Actr3*) [44] (Figure 3f; Figure S13), and inflammation‐related genes (*Tlr9, Ikbke, Stat1*) [45] (Figure 3h; Figure S13). Motif enrichment analysis of the two clusters revealed a reciprocal shift in transcription factor usage (Figure 3i; Figure S14). Rigidity‐gained regions (C1) were dominated by ETS‐family motifs, led by the microglial master regulators PU.1 and IRF8, together with MEF2 and RUNX. In contrast, rigidity‐lost regions (C2) were overwhelmingly enriched for AP‐1 (Fos/Jun) motifs, which mark basal homeostatic enhancers. Thus, matrix rigidity establishes microglial activation enhancers while dismantling the compliant‐state AP‐1 enhancer landscape, consistent with the bidirectional program switch identified above.

Collectively, these ATAC‐seq analyses indicate that matrix rigidity significantly influences chromatin accessibility through mechanotransduction pathways. The predominant distribution of DARs in non‐exonic regions demonstrates that extensive non‐coding regulatory mechanisms subsequently impact inflammatory gene activation in microglial cells through histone modification. These epigenetic alterations are driven by mechanosensing and mechanoadaptation, primarily through F‐actin cytoskeletal remodeling and the resulting nuclear deformation (illustrated in Figure S15). This remodeled chromatin landscape establishes a “mechanoprimed” state in microglial cells by redistributing accessibility—reducing it across gene‐poor regions associated with homeostatic programs while focally increasing it at promoter‐proximal inflammatory loci, while the mechanosensitive transcriptional coactivator MRTF‐A further amplifies inflammatory gene expression through enhanced nuclear translocation under rigid matrix conditions.

### 2.3 Matrix Rigidity Cooperates With LPS Signals to Amplify Microglial Activation of Inflammatory Responses

Having confirmed the phenomenon of rigidity‐mediated epigenetic priming in microglial cells for inflammatory gene activation, we next investigated the interplay between matrix rigidity and inflammatory biochemical cues. To simulate inflammation, we utilized lipopolysaccharide (LPS) as a model biochemical stimulus. BV2 cells were seeded on PAA gels of varying rigidity for 12 h before exposure to LPS (1 µg/mL) for an additional 12 h (Figure S16a). Following LPS treatment, cells exhibited a morphological transition from an elongated to a rounded state, even on rigid matrices, accompanied by reduced cell area [46, 47, 48], but maintained high F‐actin intensity (Figure S16b,c).

**FIGURE 4:**
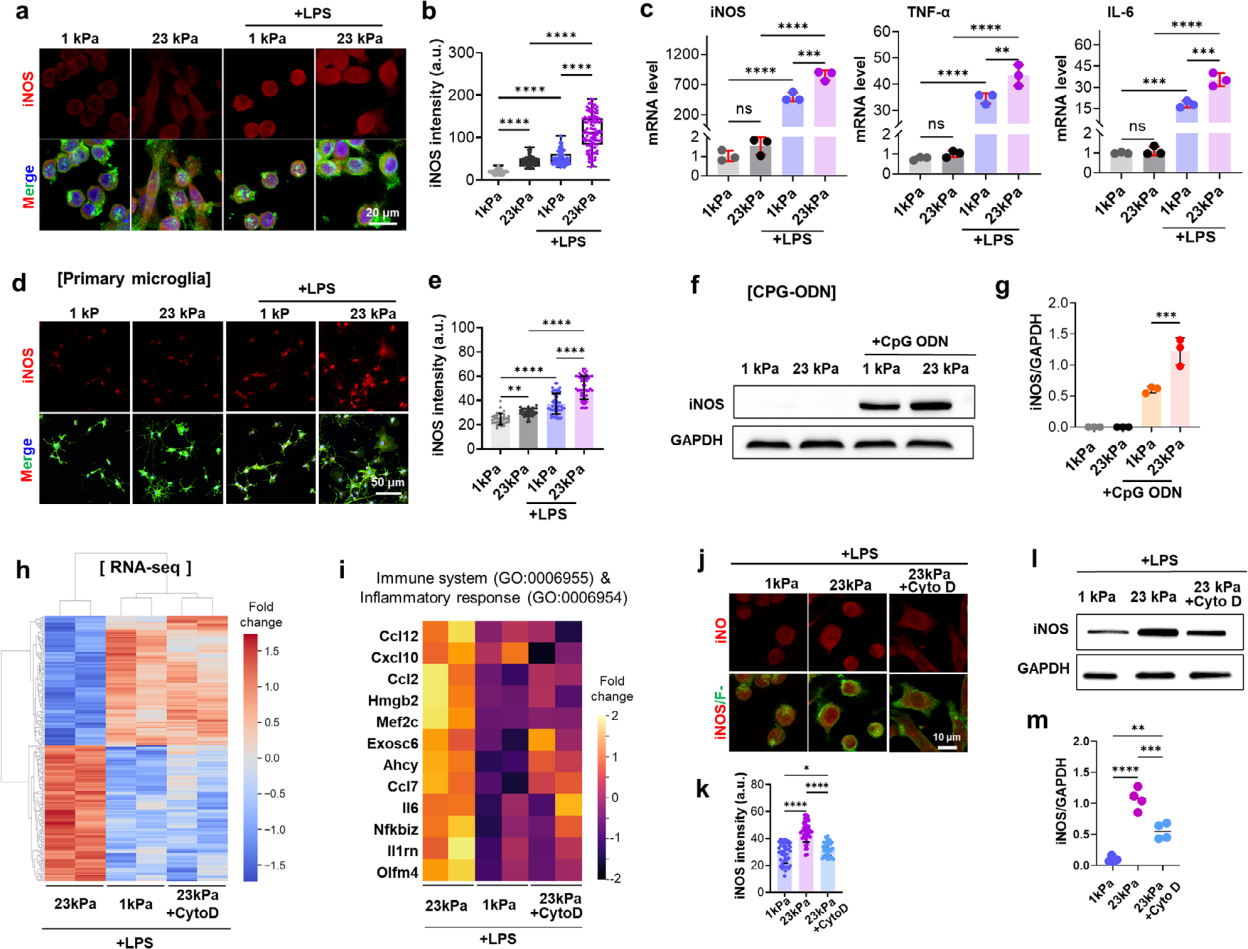
Matrix rigidity synergistically amplifies biochemically induced microglial inflammation via actin cytoskeletal tension. (a,b) Representative confocal images and semi‐quantification of iNOS expression (red) in microglial (BV2) cells cultured under varying matrix rigidities with lipopolysaccharide (LPS) treatment (*n* = 97). Cells were co‐stained for F‐actin (green) and nuclei (blue). (c) qRT‐PCR analysis of inflammatory gene expression, normalized to the unstimulated 1 kPa control condition (*n* = 3). (d,e) Validation in primary microglia from neonatal mice. Representative immunofluorescence images and semi‐quantification demonstrating that iNOS expression in primary cells follows the same rigidity‐dependent trends observed in BV2 cells (*n* = 52). (f,g) Influence of an alternative biochemical stimulus, CpG‐ODN (1 µm). Western blot analysis of iNOS expression and corresponding semi‐quantification normalized to GAPDH (*n* = 3). (h,i) Bulk RNA‐seq analysis of LPS‐treated cells cultured on 1, 23, or 23 kPa matrices with cytochalasin D (CytoD) pretreatment (1 h prior to LPS). (h) Heatmap of 797 DRGs. Notably, cytoskeletal disruption via CytoD profoundly reversed rigidity‐induced transcriptomic changes, closely mirroring the expression profile of cells on compliant matrices. (i) Analysis of inflammatory genes categorized under immune system processes (GO: 0006955) and inflammatory responses (GO: 0006954). Highly expressed genes were defined by a fold change ≥ 1.3 and *p* < 0.05 at 23 kPa compared to either 1 or 23 kPa + CytoD (*n* = 2). (j,k) Representative confocal images (iNOS, red; F‐actin, green) and semi‐quantification illustrating the suppressive effect of CytoD treatment on iNOS fluorescence intensity. (l,m) Western blot validation of CytoD‐mediated iNOS suppression, with semi‐quantification normalized to GAPDH (*n* = 4). Data are presented as mean ± SD from three or four independent biological experiments, as indicated. Sample sizes (*n*) represent the number of cells or ROIs analyzed per condition for imaging data. Statistical significance was determined using one‐way ANOVA followed by Tukey's post‐hoc test: ****p < 0.0001, ***p < 0.001, **p < 0.01, *p < 0.05; ns, not significant (*p* > 0.05).

Immunostaining for the inflammation marker iNOS (inducible nitric oxide synthase) revealed the highest fluorescence intensity in cells cultured on rigid matrices with LPS treatment, demonstrating a cooperative effect between physical (matrix rigidity) and biochemical (LPS) cues in microglial inflammatory activation (Figure 4a,b; Figure S17a). Consistently, the expression of the microglial activation marker, Iba1 (ionized calcium‐binding adaptor molecule 1) [49], mirrored that of iNOS (Figure S17b,c). Given that LPS activates Toll‐like receptors (TLRs) and downstream signaling pathways to promote inflammatory gene transcription [50, 51], we assessed the mRNA levels of key inflammatory markers (*iNOS*, *TNF‐α*, and *IL‐6*) via RT‐qPCR. The results indicated a significant upregulation of all inflammatory genes in microglia cultured on rigid matrices with LPS stimulation compared to all other conditions (Figure 4c).

To determine whether this cooperative effect of physical and biochemical cues was consistent across microglial models, we validated these findings using primary microglia from neonatal mice. Primary microglia exhibited iNOS expression patterns similar to BV2 cells in response to matrix rigidity and LPS treatment (Figure 4d,e). Additionally, when BV2 cells were exposed to another inflammatory inducer, CpG‐ODN, which targets TLR9 and its downstream signaling, similar cooperative interactions between CpG‐ODN and matrix rigidity were observed (Figure 4f,g). This suggests that matrix rigidity interplays with biochemical signals to drive microglial inflammatory activation, irrespective of the inducer type.

We next conducted bulk RNA sequencing (*n* = 2) on LPS‐treated BV2 cells across different rigidity conditions (1 kPa vs. 23 kPa), and identified 797 differentially regulated genes (DRGs; |fold change| ≥ 1.3 with *p* < 0.05) (Figure 4h). Strikingly, pharmacological inhibition of the actin cytoskeleton via CytoD profoundly reversed these rigidity‐induced changes, closely mirroring the transcriptomic profile observed on compliant matrices. This indicates that F‐actin tension is a key driver of the rigidity‐driven microglial inflammatory gene expression. Principal component analysis (PCA) confirmed distinct clustering influenced by matrix rigidity and cytoskeletal integrity, with CytoD repositioning samples from rigid‐matrix profiles toward compliant‐matrix profiles (Figure S18a). A Venn diagram revealed 215 contra‐regulated genes, common to the comparisons of 23 kPa versus 1 kPa and 23 kPa + CytoD versus 23 kPa (Figure S18b). GO analysis highlighted enriched biological processes, notably inflammatory and mechanotransduction pathways (Figure S18c). Further analysis of inflammatory genes categorized under immune system (GO: 0006955) and inflammatory response (GO: 0006954) processes identified the upregulation of several NF‐κB signaling pathway components on rigid matrices with LPS treatment, suggesting that rigidity potentiates canonical inflammatory signaling (Figure 4i).

We further assessed the expression of the key inflammatory marker iNOS. CytoD treatment significantly reduced iNOS protein levels in microglia cultured on 23 kPa with LPS, as evidenced by immunofluorescence imaging (Figure 4j,k) and Western blot analysis (Figure 4l,m). These results confirm that rigidity‐induced F‐actin cytoskeletal remodeling plays a crucial role in amplifying intracellular signaling in response to biochemical cues.

### 2.4 Matrix Rigidity and LPS Cues Accelerate NF‐κB Activation via Cytoskeletal‐Mediated MRTF‐A Translocation

**FIGURE 5:**
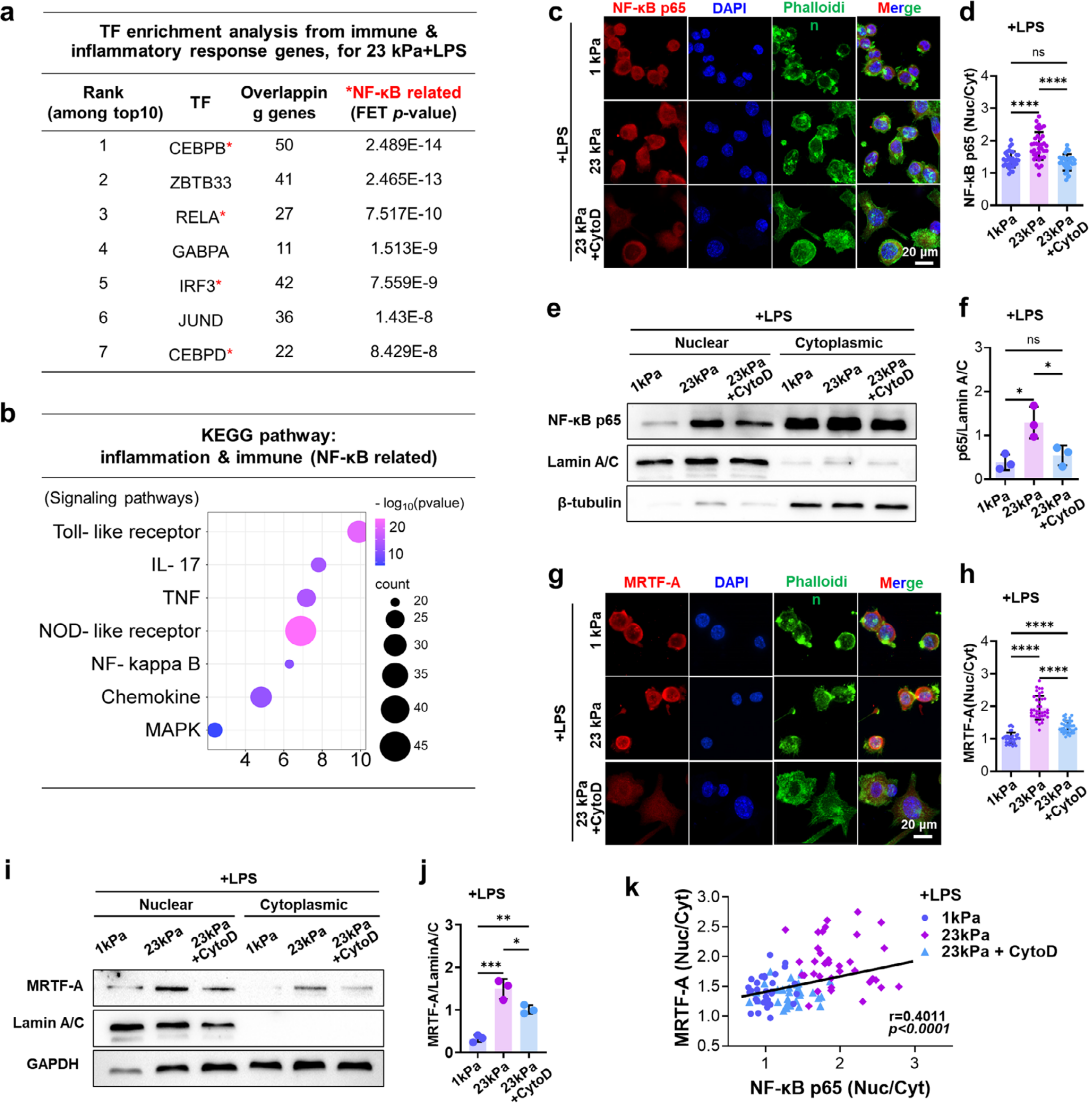
Matrix rigidity and LPS cooperatively activate NF‐κB, correlating with cytoskeletal‐mediated MRTF‐A nuclear translocation. (a) Transcriptomic analysis revealing the upregulation of immune system processes (GO: 0006955) and inflammatory responses (GO: 0006954) in cells cultured on 23 kPa versus 1 kPa under LPS treatment. Red asterisks highlight the enrichment of NF‐κB‐related transcription factors. (b) KEGG pathway analysis of the RNA sequencing data, confirming the enrichment of NF‐κB‐related signaling pathways on rigid matrices. (c,d) Representative confocal immunostaining images and semi‐quantification of NF‐κB p65, demonstrating its enhanced nuclear localization on 23 kPa versus 1 kPa under LPS stimulation, quantified via nuclear‐to‐cytoplasmic ratios (*n* = 16–20). (e,f) Western blot analysis and semi‐quantification of NF‐κB p65 expression, corroborating increased nuclear NF‐κB p65 levels at 23 kPa versus 1 kPa under LPS (*n* = 3). (g,h) Representative confocal immunostaining images and semi‐quantification of MRTF‐A, revealing enhanced nuclear localization at 23 kPa versus 1 kPa under LPS, quantified via nuclear‐to‐cytoplasmic intensity ratios (*n *= 17–21). (i,j) Western blot analysis and semi‐quantification of MRTF‐A expression, confirming elevated nuclear levels at 23 kPa versus 1 kPa under LPS. Notably, treatment with the actin polymerization inhibitor CytoD reduced MRTF‐A nuclear localization (*n* = 3). (k) Correlation analysis between NF‐κB p65 and MRTF‐A nuclear‐to‐cytoplasmic intensity ratios (*n* = 65), revealing a significant positive correlation (Pearson's *r* = 0.4011, *p* = 0.0001). Data are presented as mean ± SD from three independent biological experiments. Sample sizes (*n*) represent the number of cells or ROIs analyzed per condition for imaging data. Statistical significance was determined using one‐way ANOVA followed by Tukey's post‐hoc test: ****p < 0.0001, ***p < 0.001, **p < 0.01, *p < 0.05; ns, not significant (*p* > 0.05).

To elucidate potential transcriptional regulators influenced by both rigidity and biochemical stimuli, we conducted in silico transcriptional enrichment analysis utilizing immune and inflammatory gene sets upregulated on rigid matrices in the presence of LPS. Among the top‐ranked transcription factors, *CEBPB*, *RELA*, and *IRF3* were identified, all of which are intimately associated with the NF‐κB pathway [52] (Figure 5a), suggesting a pivotal role for NF‐κB signaling in rigidity‐amplified microglial inflammation. KEGG analysis of inflammatory genes upregulated on rigid matrices further underscored an enrichment for NF‐κB‐related pathways (Figure 5b). Examination of NF‐κB p65 activation in microglia under varying matrix rigidities revealed enhanced nuclear localization at 23 kPa compared to 1 kPa, as evidenced by immunofluorescence imaging (Figure 5c,d) and Western blot analysis (Figure 5e,f). Furthermore, when F‐actin polymerization was pharmacologically disrupted using CytoD, the increased nuclear localization of NF‐κB p65 observed on rigid matrices was reversed to levels comparable to those on the compliant matrices.

Having confirmed the crucial involvement of F‐actin cytoskeletal processes in NF‐κB p65 nuclear localization, we investigated whether MRTF‐A contributes to NF‐κB activation and signaling. The MRTF‐A/serum response factor (SRF) complex, known to be vital for NF‐κB‐dependent inflammation, is activated under conditions of high F‐actin polymerization [21]. Mechanistically, LPS promotes the NF‐κB‐dependent recruitment of MRTF‐A to inflammatory gene promoters, enhancing the reciprocal nuclear localization of NF‐κB and its transcriptional activity [53]. Rigid matrices increased MRTF‐A abundance in both the cytosol and nucleus, with a more pronounced accumulation in the nucleus. This was supported by an elevated nuclear‐to‐cytosolic MRTF‐A ratio quantified from immunofluorescence images (Figure 5g,h) and by Western blot analysis (Figure 5i,j). Similar rigidity‐driven nuclear translocation of mechanosensitive transcriptional regulators, including MRTF‐A and YAP, has been reported in diverse cellular contexts [54, 55, 56]. Furthermore, immunofluorescence imaging and Western blot analysis revealed significantly higher SRF expression and an elevated nuclear‐to‐cytoplasmic ratio at 23 kPa versus 1 kPa (Figure S19a,b). In addition, under serum‐free conditions, matrix rigidity did not affect inflammation activation, as deduced by iNOS levels, suggesting that the mechanosensitive MRTF‐A/SRF signaling pathway requires serum factors to function [57] (Figure S19c). Consistent with the reported rigidity sensitivity of YAP, immunofluorescence analysis also revealed a significantly elevated YAP nuclear‐to‐cytoplasmic ratio on rigid matrices (Figure S19d), indicating that matrix rigidity engages both mechanosensitive effectors in microglia [58]. Of note, F‐actin inhibition (+CytoD) reversed this transcriptional activation. Moreover, a positive correlation between the NF‐κB p65 and MRTF‐A nuclear‐to‐cytoplasmic ratio across conditions (Figure 5k) highlights a strong interplay between the cytoskeletal mechanosensor (MRTF‐A) and the key inflammatory transcriptional regulator (NF‐κB p65).

### 2.5 Increased Chromatin Accessibility Driven by Mechanochemical Cues Promotes Microglial Inflammatory Activation

**FIGURE 6:**
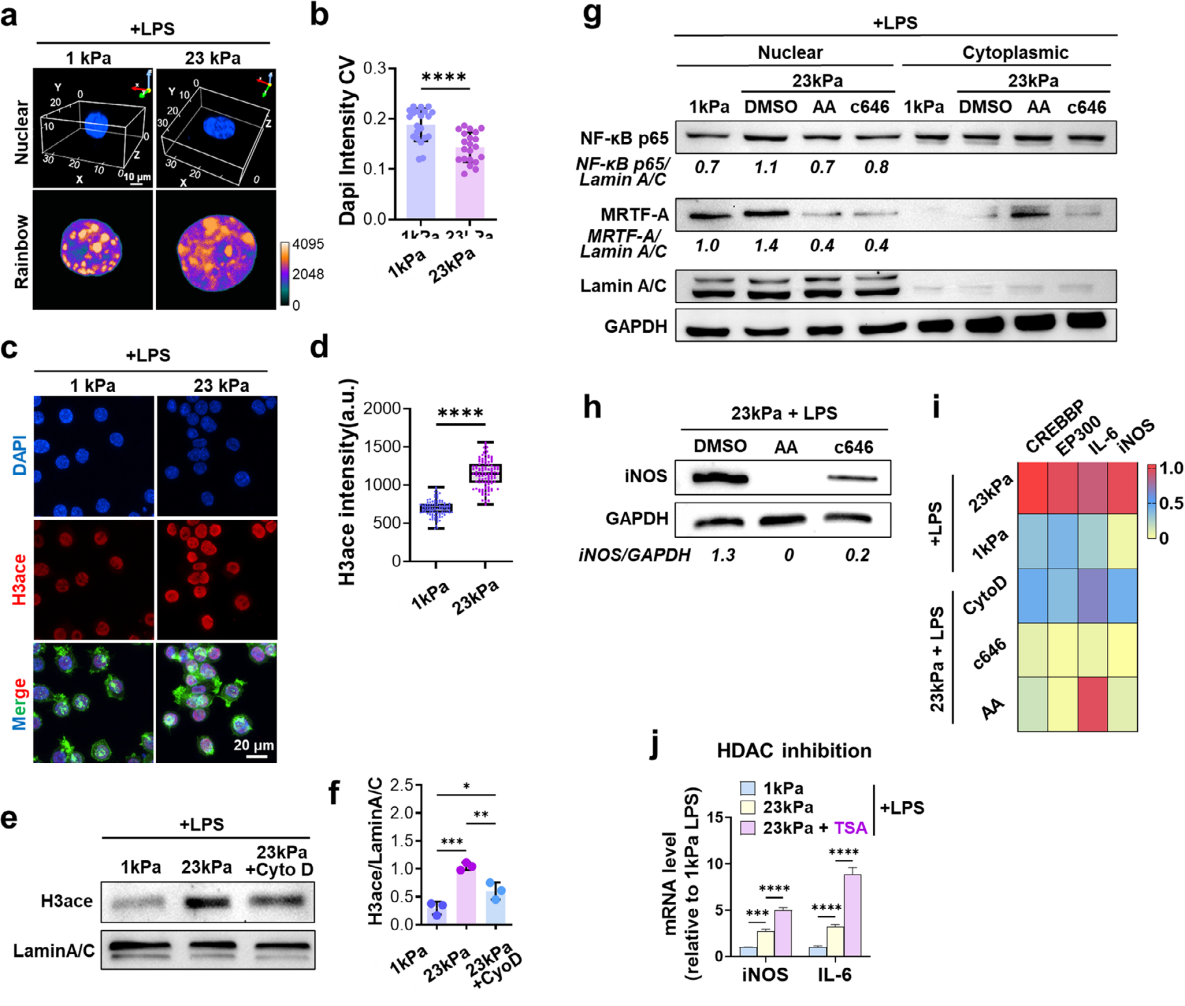
Matrix rigidity, in conjunction with biochemical signals, enhances chromatin accessibility to drive microglial inflammatory activation. (a,b) Representative high‐resolution images of DAPI‐stained microglial nuclei, displaying 3D nuclear morphology and DNA loci, cultured on compliant (1 kPa) or rigid (23 kPa) matrices under LPS stimulation. Semi‐quantification of chromatin condensation (visualized via a rainbow intensity scale) indicates that cells on 23 kPa exhibited a significantly more decondensed chromatin state compared to 1 kPa (*n* = 21). (c,d) Representative immunofluorescence images and semi‐quantification of acetylated histone H3 (H3ace) intensity, demonstrating increased levels of this representative euchromatin mark in the nuclei of LPS‐treated cells on 23 kPa versus 1 kPa (*n* = 105). (e,f) Western blot analysis and semi‐quantification of H3ace levels. Matrix rigidity increased nuclear H3ace levels, an effect that was reversed by CytoD treatment. (g) Analysis demonstrating that pharmacological inhibition of histone acetyltransferases (HATs) using C646 (20 µm) or anacardic acid (AA, 50 µm) reduced the rigidity‐induced nuclear accumulation of both MRTF‐A and NF‐κB. (h) Quantitative real‐time PCR (RT‐qPCR) analysis showing the rigidity‐mediated upregulation of inflammatory (*iNOS*, *IL‐6*) and HAT (*Ep300*, *Crebbp*) genes. Treatment with the F‐actin disruptor CytoD or the HAT inhibitors (C646 or AA) reversed this upregulation (*n* = 3). (i) Western blot analysis and semi‐quantification of iNOS protein expression. (j) RT‐qPCR analysis showing that pretreatment with the histone deacetylase (HDAC) inhibitor Trichostatin A (TSA, 30 nm) for 1 h prior to a 12 h LPS stimulation further augmented the rigidity‐driven upregulation of inflammatory genes. Expression levels were normalized to the TSA‐untreated 1 kPa control condition (*n* = 3). Data are presented as mean ± SD from three independent biological experiments. Sample sizes (*n*) represent the number of cells or ROIs analyzed per condition for imaging data. Statistical significance was determined using one‐way ANOVA followed by Tukey's post‐hoc test: ****p < 0.0001, ***p < 0.001, **p < 0.01, *p < 0.05; ns, not significant (*p* > 0.05).

We have demonstrated that microglia undergo mechanopriming toward a more open chromatin structure under rigid matrix conditions, even in the absence of biochemical inducers. We further investigated whether this phenomenon persists in the presence of biochemical inflammatory inducers. Microglia (BV2 cells) cultured on rigid matrices (23 kPa) with LPS stimulation exhibited more diffuse DAPI‐stained loci in the nucleus, as observed through high‐resolution confocal microscopy, indicating decreased chromatin condensation due to matrix rigidity (Figure 6a,b). Examination of H3ace, a key euchromatin marker, revealed a significantly increased fluorescence intensity at 23 kPa compared to 1 kPa with LPS stimulation, as shown by immunofluorescence images (Figure 6c,d) and confirmed by Western blot analysis (Figure 6e,f). These rigidity‐dependent decreases in nuclear condensation and increases in H3ace were also observed in primary microglia exposed to LPS (Figure S20a–d), indicating that mechanosensitive chromatin remodeling under LPS stimulation is conserved across microglial models. Moreover, pharmacological inhibition of the actin cytoskeleton (+CytoD) in BV2 cells significantly attenuated the rigidity‐induced increase in H3ace, suggesting that F‐actin cytoskeletal remodeling mediates this chromatin alteration. These results demonstrate that matrix rigidity remodels the microglial chromatin structure toward more open configurations (primarily via increased histone acetylation) in the presence of the biochemical inflammatory inducer LPS, with this modulation mediated through the F‐actin cytoskeletal network. This chromatin modification likely influences downstream microglial inflammatory responses.

We thus sought to investigate whether histone acetylation serves as a key regulator of microglial inflammatory responses. We employed pan‐histone acetyltransferase (HAT) inhibitors (C646 and anacardic acid, AA) [59, 60], which significantly reduced H3ace levels and reversed rigidity‐induced chromatin decondensation back to a condensed state similar to that observed on compliant matrices or under F‐actin inhibition (+CytoD) (Figure S21a–d) [61]. Importantly, the pharmacological blockade of histone acetylation substantially reduced the nuclear translocation (and nuclear retention) of both NF‐κB and MRTF‐A, as demonstrated by Western blot analysis (Figure 6g). Moreover, iNOS protein levels were also significantly suppressed upon HAT inhibition as determined by Western blot analysis (Figure 6h). Indeed, the expression of inflammatory genes (*iNOS* and *IL‐6*), which was elevated on rigid matrices with LPS stimulation, was markedly downregulated by HAT inhibitor treatment, alongside decreased expression of HAT genes (*Ep300* and *Crebbp*) (Figure 6i; Figure S22). Collectively, these findings underscore the crucial interplay between F‐actin cytoskeletal organization and histone acetylation in controlling microglial inflammatory activation, whereby matrix rigidity induces F‐actin‐dependent chromatin remodeling that modulates the transcriptional activities of MRTF‐A and NF‐κB by regulating chromatin accessibility at inflammatory gene loci.

We further explored whether enhancing histone acetylation augments microglial inflammation. To investigate this, we inhibited histone deacetylases (HDACs) using Trichostatin A (TSA) [62] in cells cultured under rigid matrix conditions (23 kPa) with LPS stimulation. Remarkably, inflammatory genes *(iNOS* and *IL‐6*) were highly upregulated with TSA treatment, in the sequence of 23 kPa < 23 kPa with LPS < 23 kPa with LPS + TSA (Figure 6j). These results suggest that chromatin modification, particularly histone acetylation, serves as a key regulator in amplifying inflammatory responses in microglial cells under mechanochemical stimuli (rigid matrix and the inflammatory inducer LPS).

To extend these findings and explore their broader implications, we investigated the functional impacts of rigidity‐induced microglial inflammatory activation, particularly its effects on astrocytes (Figure S23a). Primary astrocytes, cultured in conditioned medium from microglia (BV2) precultured on varying matrix rigidities along with LPS, exhibited significant morphological changes indicative of reactive astrocytes [63], especially when exposed to conditioned medium from the rigid matrix conditions (Figure S23b,c). Furthermore, proinflammatory genes (*TNF‐α, IL‐6*) significantly increased in astrocytes treated with microglia‐conditioned medium, mirroring the morphological changes (Figure S23d). Astrocyte viability also decreased significantly upon conditioned medium treatment (Figure S23e), suggesting that rigidity‐ and LPS‐driven microglial inflammatory activation drives astrocytes toward elevated inflammatory activity.

### 2.6 Regulation of Inflammatory Genes Through MechanoCRE‐Mediated 3D Long‐Range Chromatin Interactions

The mammalian genome is hierarchically organized within the nucleus, with multi‐layered structures that are tightly linked to gene regulation [64, 65, 66, 67]. Active and inactive genomic regions are spatially compartmentalized into A/B compartments, and further subdivided into topologically associating domains (TADs) [64, 65]. Within these higher‐order structures, *cis*‐regulatory elements (CREs) engage in long‐range interactions with their target genes [68, 69]. To investigate how matrix rigidity affects higher‐order chromatin structures and subsequently regulates gene expression, we performed in situ Hi‐C in LPS‐treated BV2 cells under 1 and 23 kPa conditions (Figure S24a). Following rigorous quality control, we captured 408 and 345 million long‐range (>20 kb) *cis*‐interactions for the 1 and 23 kPa conditions, respectively (Table S1). We first examined the dynamics of A/B compartments and TADs in LPS‐treated microglia, but found no significant alterations in compartmentalization (on the multi‐megabase scale) or TAD structures (on the megabase scale) in response to matrix rigidity (Figure S24b–d).

**FIGURE 7:**
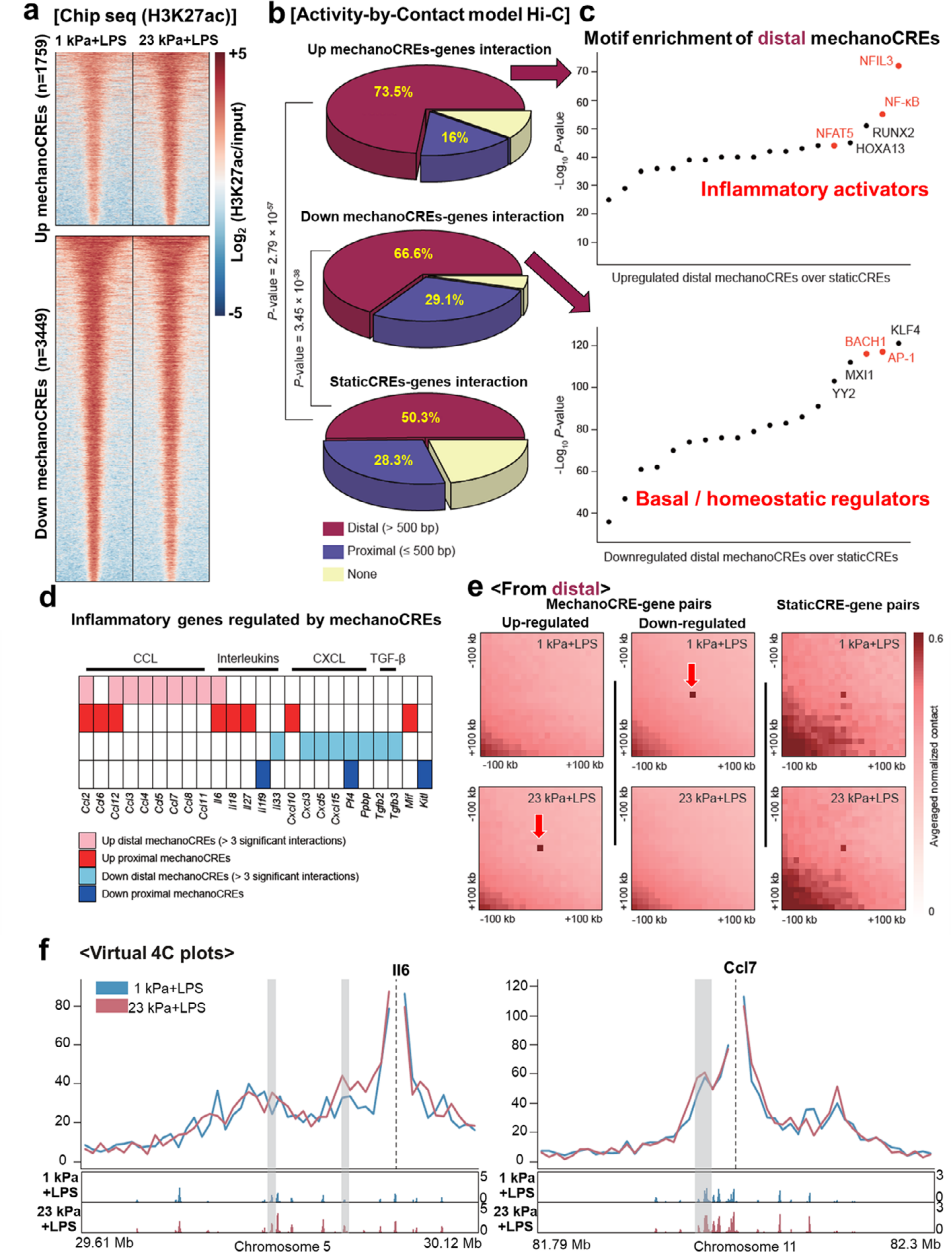
Matrix rigidity employs distal mechanoCREs and long‐range interactions to regulate gene expression. (a) H3K27ac enrichment at mechanoCREs. Tornado plots illustrating ±5 kb regions around upregulated (23 > 1 kPa, *FDR* < 0.05, 1759, *n* = 3) and downregulated (1 > 23 kPa, *FDR* < 0.05, 3449, *n* = 3) mechanoCREs in LPS‐treated BV2 cells under 1 and 23 kPa conditions. Colors represent the log_2_ H3K27ac ChIP‐seq signal relative to the input signal. (b) Promoter‐distal enrichment of mechanoCREs. Pie charts comparing the percentages of promoter‐distal CREs and promoter‐proximal CREs within upregulated mechanoCREs, downregulated mechanoCREs, and static CREs. Static CREs (*n* = 3077) were defined as highly stable CREs showing no significant change in H3K27ac between conditions (*FDR* ≥ 0.95), selected using a stringent threshold to ensure a robust control set. CREs were categorized as promoter‐proximal (within 500 bp of a TSS), promoter‐distal (>500 bp from TSS but with ABC score > 0.02), or unclassified (neither proximal nor significantly linked). *P*‐values were calculated using a two‐sided Fisher's exact test. (c) Motif enrichment analysis of upregulated (top) and downregulated (bottom) distal mechanoCREs relative to static CREs. Transcription factor motifs overrepresented in promoter‐distal mechanoCREs are ranked by enrichment. The top five enriched motifs are highlighted, and motifs corresponding to immune‐related regulators are highlighted in red. (d) Heatmap illustrating cytokines and chemokines potentially regulated by mechanoCREs. Genes were considered potentially regulated by mechanoCREs if they were linked to more than three distal mechanoCREs showing the same direction of change (upregulated or downregulated), or if their promoters overlapped promoter‐proximal mechanoCREs. Genes that were not expressed under either condition were excluded. (e) Rigidity‐dependent CRE‐gene interactions at distal mechanoCREs. Aggregated Hi‐C contact maps showing averaged contacts within ±100 kb of CRE‐target gene pairs for upregulated distal mechanoCREs (left), downregulated distal mechanoCREs (middle), and distal static CREs (right) in LPS‐treated BV2 cells under 1 kPa (top) and 23 kPa (bottom). (f) Increased long‐range contacts at inflammatory gene loci. Virtual 4C plots highlighting increased CRE‐target gene interactions involving *Il6* (left) or *Ccl7* (right) and mechanoCREs. Normalized contact frequencies to the target genes (top) and normalized H3K27ac signal (bottom) from LPS‐treated BV2 cells under 1 kPa (blue) and 23 kPa (red) conditions are shown. Vertical dashed lines indicate the locations of the target genes, and the asterisks mark distal mechanoCREs. Vertical gray shadings highlight distal mechanoCREs with increased contacts to the target genes.

Given that higher‐order chromatin structure was stably preserved across different matrix rigidity conditions, we next asked whether matrix rigidity instead altered CRE activities, thereby affecting gene expression. To test this hypothesis, we performed H3K27ac chromatin immunoprecipitation followed by high‐throughput sequencing (ChIP‐seq), a mark of active *cis*‐regulatory elements (CREs), in LPS‐treated microglial cells cultured under different matrix rigidity conditions (1 vs. 23 kPa). Differential analysis of H3K27ac‐peaked regions identified 1759 upregulated rigidity‐responsive CREs (23 kPa > 1 kPa, *FDR* < 0.05) and 3449 downregulated rigidity‐responsive CREs (1 kPa > 23 kPa, *FDR* < 0.05), which we collectively termed ‘mechanoCREs’ (Figure 7a; Figure S24e). The larger number of downregulated mechanoCREs is consistent with previous reports that mechanical stress can induce downregulation of active chromatin regions [70]. To identify the putative target genes of these mechanoCREs, we employed an integrated approach combining Hi‐C and the activity‐by‐contact (ABC) model to predict potential regulatory target genes (Figure S24f) [71]. The ABC model predicts target genes by integrating CRE activity (measured by H3K27ac levels) with interaction frequencies captured by Hi‐C. Of the 49 716 total CREs analyzed, 10 755 and 10 752 were promoter‐proximal (<500 bp from the transcription start site) in LPS‐treated BV2 cells under 1 and 23 kPa conditions, respectively. In contrast, 22 787 and 24 073 were promoter‐distal CREs predicted to target specific genes under the corresponding conditions (Figure S24f).

Notably, both upregulated and downregulated mechanoCREs were frequently classified as promoter‐distal CREs compared with static CREs (*n* = 3077), which were defined as highly stable CREs showing no significant change in H3K27ac (*FDR* ≥ 0.95), selected using a stringent threshold (Figure 7b). These distal mechanoCREs were enriched for distinct immune regulators relative to static CREs: upregulated mechanoCREs were enriched for NFIL3, NF‐κB, and NFAT5 binding sites, whereas downregulated mechanoCREs were enriched for BACH1 and AP‐1 binding sites (Figure 7c, Table S2). These two sets of regulators have opposing relationships to inflammation. NFIL3, NF‐κB, and NFAT5 are inducible activators of macrophage inflammatory transcription that are engaged downstream of Toll‐like receptor signaling [72], consistent with their enrichment at loci that gain activity on rigid matrices. In contrast, BACH1 is a heme‐regulated transcriptional repressor that maintains macrophages in a resting state and restrains premature inflammatory gene expression [73]; its enrichment at loci that lose activity on rigid matrices therefore suggests that stiffening withdraws a repressive, homeostatic regulatory program. Thus, matrix rigidity does not simply amplify inflammation but reciprocally rewires the regulatory landscape—engaging inducible activators while disengaging homeostatic repressors. Direct comparison between upregulated and downregulated mechanoCREs further supported that NFIL3 was preferentially enriched in upregulated mechanoCREs, while BACH1 was preferentially enriched in downregulated mechanoCREs (Figure S24g, Table S2). In contrast, promoter‐proximal mechanoCREs showed weaker enrichment for immune regulator binding sites, underscoring the central role of promoter‐distal regulatory elements in mediating matrix rigidity‐induced immune responses (Figure S24h, Table S2).

Given the strong association between immune regulator binding sites and mechanoCREs, we next investigated cytokine and chemokine genes potentially regulated by these mechanoCREs because of their central roles in immune responses. Distinct cytokine and chemokine gene families were linked to upregulated versus downregulated mechanoCREs. The upregulated set was enriched for activation‐associated regulators (NF‐κB, NFAT5) and mediators (CCL chemokines, Il6, Cxcl10), consistent with inflammatory priming [74, 75, 76]. The downregulated set included both homeostatic regulators—the repressor BACH1, TGF‐β family genes that maintain microglial identity, and the anti‐inflammatory Il19—and a distinct subset of inflammatory chemokines (Cxcl3, Cxcl5) [77, 78, 79]. Thus, rather than uniformly amplifying or suppressing inflammation, matrix rigidity qualitatively repartitions the inflammatory program, simultaneously engaging activation‐associated enhancers and disengaging both homeostatic and a subset of alternative inflammatory enhancers. These regulatory associations were more frequent for distal than for promoter‐proximal mechanoCREs, again highlighting the importance of distal regulation in immune responses (Figure 7d). Together, these findings point to a mechanism in which matrix rigidity reciprocally reprograms inflammatory gene regulation through promoter‐distal regulatory elements—engaging enhancers of activation‐associated genes while disengaging those of homeostatic and alternative inflammatory programs—acting via upstream immune transcriptional regulators.

Finally, we examined long‐range chromatin interactions between mechanoCREs and their target genes at 10 kb resolution to determine whether CRE–gene interactions are rewired alongside mechanoCRE activity. We observed rigidity‐dependent rewiring of chromatin interactions between distal mechanoCREs and their target genes (Figure 7e), and these rigidity‐dependent interactions spanned significantly greater distances than typical (static) CRE‐mediated interactions, highlighting the unique capacity of mechanoCREs to engage super‐distal genes (Figure S24i). At the Il6 and Ccl7 loci, for example, inflammatory genes activated by matrix rigidity under LPS treatment showed increased long‐range interactions with distal mechanoCREs (Figure 7f). Together, these results demonstrate that matrix rigidity reshapes inflammatory responses not only by altering mechanoCRE activity but also by rewiring the long‐range chromatin interactions between these elements and their target genes.

## 3 Conclusions

**FIGURE 8:**
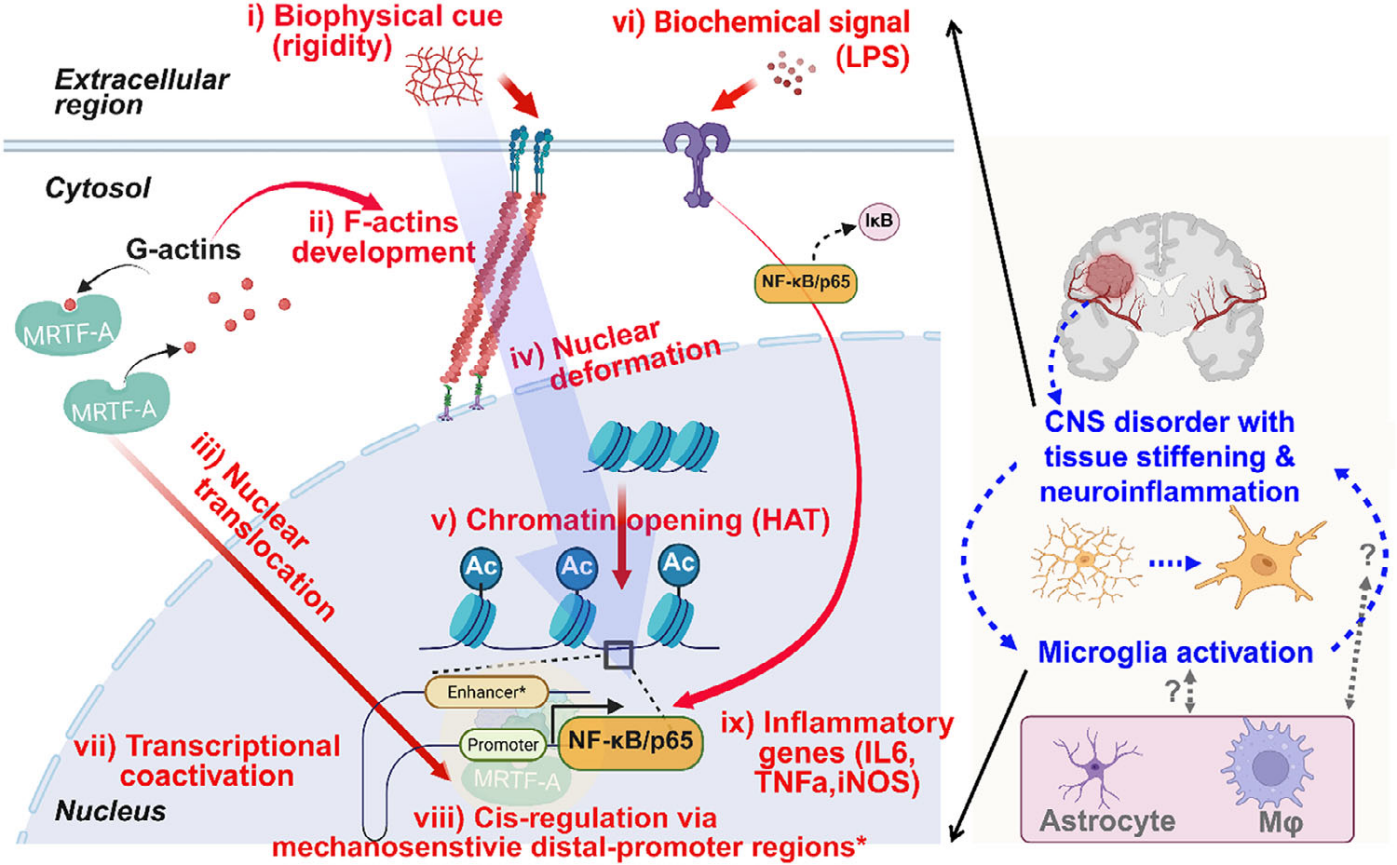
Schematic illustration of the mechanochemical cascade driving microglial inflammatory activation. Microglia mechanosense the biophysical cue of matrix rigidity (i), initiating progressive actin cytoskeletal remodeling (ii). This facilitates the nuclear translocation of the key mechanosensor MRTF‐A (iii) while inducing nuclear deformation (iv). Consequently, nuclear chromatin undergoes decondensation (v) through increased histone acetylation. These events mechanoprime microglia, achieving enhanced accessibility at specific genomic loci. Concurrently, biochemical signals such as LPS (vi) activate the inflammatory signaling cascade, primarily through the inflammatory master regulator NF‐κB (vii). Upon nuclear translocation, MRTF‐A serves as a transcriptional coactivator with NF‐κB (viii) to exert *cis*‐regulatory control through promoter‐distal mechanosensitive elements (mechanoCREs; ix), thereby upregulating pro‐inflammatory genes including *IL‐6*, *TNF‐α*, and *iNOS*. These mechanoadaptive phenomena in microglia are implicated in pathological neural tissue stiffening (shown on the right side in the yellow panel). The activated microglia secrete inflammatory cytokines that influence neighboring astrocytes to become reactive, contributing to a chronic neuroinflammatory microenvironment. Whether this neuroinflammation further exacerbates matrix stiffening through extracellular matrix deposition—creating a self‐reinforcing mechanobiological feedback loop—remains to be elucidated in future studies.

This study identifies matrix rigidity as a key regulator of microglial inflammatory activation through a coordinated mechanotransduction cascade that involves actin cytoskeletal remodeling, nuclear deformation, and epigenetic reprogramming (Figure 8). We demonstrate that microglia mechanosense elevated rigidity of matrices, leading to actin cytoskeletal rearrangement, nuclear flattening, and broad chromatin decompaction via increased histone acetylation, accompanied by a bidirectional redistribution of chromatin accessibility in which homeostatic loci close and inflammatory regulatory elements open. These mechanoadaptive changes facilitate the nuclear translocation of MRTF‐A and cooperate with biochemical inducers to amplify NF‐κB signaling, collectively driving the transcription of inflammatory genes. Epigenomic profiling reveals that rigidity‐dependent transcriptional activation is largely mediated through distal *cis*‐regulatory elements, termed mechanoCREs, which form de novo long‐range chromatin interactions enriched for the binding motifs of immune‐related transcription factors. These mechanistic insights highlight how aberrant neural tissue stiffening, often arising from brain injury, aging, or neurodegenerative conditions, can act as a primary physical trigger for chronic neuroinflammation. In such pathological contexts, microglial mechanosensing may initiate a detrimental, self‐reinforcing cycle: tissue rigidity drives microglial inflammatory activation, leading to the secretion of cytokines that further promote extracellular matrix deposition and subsequent tissue stiffening. Taken together, our results uncover a fundamental mechanobiological pathway by which matrix rigidity modulates neuroinflammation, suggesting potential therapeutic strategies targeting mechanotransduction and epigenetic remodeling to break the cycle of chronic neuroinflammation in CNS disorders involving pathological tissue stiffening.

### 3.1 Limitations of the Study

While this study establishes a mechanistic link between matrix rigidity and microglial inflammatory activation through actin cytoskeletal remodeling, chromatin accessibility, and mechanoCRE‐mediated transcriptional regulation, several limitations remain. First, the findings are primarily based on the BV2 microglial cell line and primary murine microglia, which, while widely used, may not fully capture the physiological behavior of primary ‘human’ microglia. Second, the matrix models employed, though well‐controlled, do not replicate the complexity of the in vivo neural microenvironment, including its 3D architecture, biochemical gradients, and cellular heterogeneity. Specifically, our 2D polyacrylamide gel system shows predictable vertical nuclear flattening that facilitated mechanistic dissection, but the geometric aspects of nuclear deformation may differ in 3D tissue environments where deformation patterns could be more heterogeneous depending on local matrix architecture and cell–cell contacts. Third, while the pharmacological inhibition of actin polymerization and histone acetylation attenuated microglial activation in vitro, the therapeutic relevance of these interventions needs validation with appropriate in vivo models of chronic neuroinflammation. Future work integrating human microglia, organotypic models, and in vivo approaches will be crucial for strengthening the validation and translational relevance of these findings in the context of CNS disorders.

## 4 Materials and Methods

### 4.1 Preparation of Polyacrylamide Gels

Polyacrylamide (PAA) gel substrates with varying rigidity levels were prepared following an adapted protocol from a previous study [80]. Briefly, round glass coverslips (25 mm or 15 mm diameter; 111650, Marienfeld) were sequentially treated with 0.1 N NaOH, silanized using 3‐aminopropyl triethoxysilane (281778, Sigma), and coupled with glutaraldehyde to create a protein‐crosslinkable surface. PAA solutions were made from 40% acrylamide and 2% bis‐acrylamide stocks (1610140 and 1610142, Bio‐Rad) in deionized water (ddH_2_O), then degassed in a desiccator for at least 30 min. Polymerization was initiated by adding 10% tetramethyl ethylenediamine (TEMED) and 10% ammonium persulfate (APS) (T9281 and A3678, Sigma), forming hydrogels with elastic moduli ranging from 1 to 100 kPa. For the crosslinking of adhesion proteins, a heterobifunctional crosslinker, sulfo‐SANPAH (sulfosuccinimidyl 6 (4’‐azido‐2’‐nitrophenyl‐amino) hexanoate, F2006, Sigma), was employed to bind extracellular matrix molecules to the gel surface. Approximately 0.5 mg/mL of sulfo‐SANPAH was dissolved in H2O, and 280 µL of this solution was pipetted onto the gel surface. The PAA gel was then positioned 2.5 inches under a 365‐nm UV light source and irradiated for 10 min, followed by washing three times with 50 mm HEPES at pH 8.6. After aspirating the HEPES solution, 200 µL of 0.05 mg/mL fibronectin solution (F0895, Sigma) was pipetted onto the PAA gel and incubated overnight at 37°C.

### 4.2 Dynamically Stiffening Methacrylated Hyaluronic Acid Gels

The synthesis of methacrylated hyaluronic acid (MeHA) involved the direct immobilization of the methacrylate group from methacrylic anhydride (64100, Sigma) onto the hydroxyl group of hyaluronic acid (HA). The HA was sourced from Contipro Inc. in Borehamwood, UK, and distributed by ProTec Ingredia Ltd. The procedure followed established protocols. Briefly, sodium hyaluronate (NaHA) with a molecular weight between 25 and 45 kDa was dissolved in distilled water (2% w/v) overnight. Methacrylic anhydride was then added to the solution, and the reaction proceeded for 6 h within a temperature range of 1°C–4°C. Maintaining the pH within the range of 8 to 9 was achieved by gradually adding sodium hydroxide (5 m, Sigma). The resulting solution underwent filtration, followed by dialysis against distilled water (MWCO 12–14 kDa) at 4°C, with water changes three times per day for seven days. The polymer solution was subsequently filtered, frozen at −80°C, and freeze‐dried at −40°C for seven days. The resulting white cottony material was stored at −20°C for future use.

MeHA hydrogels with dynamic stiffening properties were prepared by two‐step photo‐polymerization of a MeHA solution. Initially, the MeHA solution was prepared using 3% (w/v) MeHA polymer dissolved in a Tris‐HCl buffer containing 1 mg/mL lithium phenyl‐2,4,6‐trimethylbenzoylphosphinate (LAP, 900889, Sigma) photoinitiator. Thiolated RGD‐containing peptide (RGD‐SH, GCGYGRGDSPG, Peptron, Republic of Korea) was incorporated at a concentration of 1 mm into the MeHA solution for 2 h at room temperature (RT) to enhance cell adhesion. To maintain a thin platform suitable for imaging and cell culture, a ∼100 µm layer of MeHA gel was created by photo‐crosslinking 19 µL of MeHA solution between two cover glasses (a bottom methacrylated coverslip and a top untreated 15 mm coverslip). Soft (∼2–3 kPa) platforms were achieved by applying 20 s of UVA exposure (OmniCure 320–390 nm wavelength) at 350 µW/cm^2^. Subsequently, cells were seeded on the compliant gels for 12 h. The compliant gels were then exposed to an additional 60 s of UVA exposure (OmniCure 320–390 nm wavelength) at 350 µW/cm^2^ to create rigid gels with a modulus of 20 kPa.

### 4.3 Cell Culture

Murine microglia cell line (BV2) was obtained from ATCC (American Type Culture Collection). Primary microglia and astrocytes were isolated from neonatal C57BL/6 mice at postnatal days 0–2 following an established protocol [81] approved by the Dankook University Animal Care Committee (DKU‐19‐016, Cheonan, Korea). Briefly, cerebella were dissected, enzymatically dissociated into a single‐cell suspension, and plated on poly‐*D*‐lysine (0.1 mg/mL) coated 60 mm dishes in high glucose DMEM supplemented with 10% fetal bovine serum and 1% penicillin‐streptomycin. After 3 days, the cell debris and medium were removed, and the culture medium was replenished to allow for microglial proliferation. All cells were cultured in high glucose DMEM (LM 001–05, Welgene), 10% fetal bovine serum (35‐015‐CV, Corning), and 1% Penicillin‐Streptomycin (15140163, Gibco) at 37°C inside a standard cell culture incubator containing 5% CO_2_.

### 4.4 Inhibitor Study

Cell viability upon inhibitor treatment was evaluated using the CCK‐8 assay. Microglial cells were seeded in 96‐well plates and treated with various concentrations of inhibitors for 12 h after 12 h of seeding. CCK‐8 reagent (Sigma) was added for the final 2 h, and absorbance was measured at 450 nm. To investigate the roles of the cytoskeleton, histone acetyltransferases (HATs), and histone deacetylases (HDACs), cells were pretreated with inhibitors for 1 h after 11 h of culture on substrates of defined rigidity, followed by LPS stimulation for 12 h in the continued presence of inhibitors. The inhibitors used were: cytochalasin D (1.5 µm, actin polymerization; 1233, Tocris Bioscience), C646 (20 µm, p300/CBP HATs; 4200, Tocris Bioscience), anacardic acid (50 µm, HATs; A7236, Sigma), trichostatin A (30 nm, class I/II HDACs; 1406, Tocris Bioscience), and CTPB (2.5 µm, p300 HAT activator; EPS001, Sigma) (Table S3).

### 4.5 Transmission Electron Microscopy

Cells were cultured on 1 and 23 kPa PAA gels for 12 h, and then washed with PBS. Cells were fixed with a solution containing 2% paraformaldehyde and 2% glutaraldehyde in PBS for 1 h at RT, and then collected into microtubes. The cell pellets were centrifuged at 1000 rpm for 3 min at RT and kept at 4°C for 12 h for further fixation. After rinsing with 0.1 m phosphate buffer, the pellets were dehydrated through a series of acetone washes (50%, 70%, 90%, 96%, and 100%; 10 min each). Following dehydration, resin‐embedded blocks were polymerized at 60°C for 48 h and sectioned into approximately 60 nm using a UC7 ultramicrotome. The sections were then stained with 5% uranyl acetate for 10 min and lead citrate for another 10 min before imaging on a Tecnai Spirit transmission electron microscope equipped with a LaB6 cathode and 1376 × 1024‐pixel CCD camera at 120 kV. Analysis of nuclear pore morphology was conducted on cross‐sections where the nuclear basket was visible. The length of nuclear pores was measured from one end of the double nuclear membrane bilayer where the basket begins to the other end where the basket terminates.

### 4.6 Immunofluorescence Analysis

Cells cultured on PAA gels were fixed with 4% paraformaldehyde (BPP‐9004, Tech & Innovation) and permeabilized with 0.1% Triton X‐100 in PBS for 10 min. After washing three times in PBS, cells were blocked with 1% bovine serum albumin (SM‐BOV‐100, GeneAll) for 1 h at RT. Primary antibodies (details provided in Table S4) were diluted in 1% BSA in PBS and applied overnight at 4°C, including rabbit anti‐Iba1, Lamin A/C, MATF‐A, iNOS, NF‐κB p65, H3ace. Following PBS washing, cells were incubated for 1 h at RT with rhodamine (TRITC)‐conjugated secondary antibodies (donkey anti‐rabbit IgG or donkey anti‐mouse IgG, Jackson ImmunoResearch) and then for 30 min with Alexa Fluor 488‐phalloidin to visualize F‐actin, and Hoechst 33342 for 5 min to label nuclei. Immunofluorescence images were acquired and analyzed using ImageJ software (http://imagejdocu.tudor.lu/) to quantify microglial morphological parameters and changes in fluorescence intensity across experimental conditions upon LPS stimulation. Nuclear rainbow images were quantified by calculating the coefficient of variation (CV) of the DAPI fluorescence intensity (nuclear condensation = standard deviation / mean intensity). A lower CV indicates more uniform DAPI staining and greater chromatin decondensation, whereas a higher CV reflects increased chromatin condensation.

### 4.7 Western Blot Analysis

Cells were lysed in RIPA buffer (EBA‐1149, ELPISBIO) supplemented with protease inhibitor cocktail (78444, ELPISBIO) for 30 min on ice. Cleared lysates were obtained by centrifugation at 12 000 rpm for 20 min at 4°C. Total protein was quantified by BCA assay (23225, Life Technologies), and equal amounts were resolved by SDS‐PAGE on 10% or 18% polyacrylamide gels, followed by transfer onto nitrocellulose membranes (1620112, Bio‐Rad). Membranes were blocked in 5% BSA‐TBST for 1 h at RT, then incubated overnight at 4°C with primary antibodies against targets of interest. After a 1× TBST wash, membranes were incubated for 1 h with secondary antibodies: goat anti‐rabbit and goat anti‐mouse coupled with HRP (horseradish peroxidase) (1: 10 000, #7074 and #7076, respectively; Cell Signaling). Visualization was performed using SuperSignal West Pico PLUS Chemiluminescent substrate (34580; Thermo Scientific). Band intensity was quantified using ImageJ software, and the results were normalized to the mean value of the control condition.

### 4.8 Quantitative Real‐Time Polymerase Chain Reaction (qRT‐PCR)

Total RNA was isolated from cells using the Ribospin^TM^ kit (304‐150, GeneAll, Seoul, South Korea) and purity was assessed by NanoDrop8000 spectrophotometry. cDNA was synthesized from 1 µg of total RNA using the AccuPower RT‐PCR PreMix (K‐2043, Bioneer) according to the manufacturer's instructions. qRT‐PCR was performed on a Veriti 96‐well thermal cycler (HID Veriti 96‐Well Thermal Cycler, 447907, Applied Biosystems, Singapore) with the following cycling conditions: 95°C for 10 s, 60°C for 30 s, 72°C for 30 s, for 40 cycles. Relative mRNA expression levels were calculated by the 2^−ΔΔ^ *^Ct^* method after normalization to β‐actin as the internal reference control. Primer sequences are listed in Table S5.

### 4.9 RNA Sequencing

For quantitative sequencing, cells were cultured on 1 and 23 kPa gels or pre‐treated with CytoD for 1 h on 23 kPa substrate. According to the manufacturer's protocol, total RNA was collected using Ribospin^TM^ (304‐150, GeneAll, Seoul, South Korea). RNA purity was determined by assaying 1 µL of total RNA extract on a NanoDrop8000 spectrophotometer. Quantitative sequencing was then performed by E‐biogen Inc. (South Korea). In brief, library preparation and sequencing were first performed. The construction of the library was carried out using the QuantSeq 3’ mRNA‐Seq Library Prep Kit (Lexogen, Inc., Austria) for control and test sample RNAs according to the manufacturer's instructions. An oligo‐dT primer containing an Illumina‐compatible sequence at its 5’ end was hybridized to the RNA (each 500 ng total RNA), and reverse transcription was performed. After degradation of the RNA template, second‐strand synthesis was initiated by a random primer containing an Illumina‐compatible linker sequence at its 5’ end. Magnetic beads were used to remove all reaction components to purify the double‐stranded library. The library was then amplified to add the complete adapter sequences required for cluster generation. The finished library was purified from PCR components. High‐throughput sequencing was performed as single‐end 75‐bp sequencing using NextSeq 500 (Illumina, Inc., USA).

Subsequently, QuantSeq 3’ mRNA‐Seq reads were aligned using Bowtie2 (Langmead and Salzberg, 2012). Bowtie2 indices were either generated from genome assembly sequences or representative transcript sequences to align to the genome and transcriptome. The alignment file was used for assembling transcripts, estimating their abundances, and detecting the differential expression of genes. DEGs were determined based on counts from unique and multiple alignments using coverage in bedtools (Quinlan AR, 2010). The read count (RC) data were processed based on the TMM+CPM normalization method using EdgeR within R (R Development Core Team, 2020) using Bioconductor (Gentleman et al., 2004). The raw data were manually analyzed using ExDEGA software (v1.6.7, E‐biogen). Gene classification was further analyzed in the public databases (DAVID (http://david.abcc.ncifcrf.gov/) and Medline databases (http://www.ncbi.nlm.nih.gov/). The QuantSeq data are available at the NCBI Gene Expression Omnibus with accession number GSE217049 (http://www.ncbi.nlm.nih.gov/geo/info/linking.html).

### 4.10 Assay for Transposase‐Accessible Chromatin Using Sequencing (ATAC‐Seq)

ATAC‐seq was conducted on microglial cells cultured on 1 and 23 kPa gels for 12 h according to the protocol described by Reference [82]. Briefly, a total of 50 000 fresh cells were washed once with 50 µL of cold PBS and resuspended in 100 µL of lysis buffer [10 mm Tris–HCl (pH 7.4), 10 mm NaCl, 0.1% IGEPAL CA‐630 and 3 mm MgCl_2_] for 10 min on ice to prepare the nuclei. After lysis, the suspension of nuclei was centrifuged at 500 g at 4°C for 10 min to remove the supernatant. The nuclei were then incubated with 50 µL of Tn5 transposition reaction mix (Illumina, USA) at 37°C for 30 min. The reaction was stopped by adding the stop buffer directly into the reaction to end the tagmentation. DNA was purified using the QIAquick PCR Purification Kit (QIAGEN, Germany). GREAT analysis (version 4.0.4) with more accessible regions in 23 than 1 kPa was performed with the default settings with whole genome background [38, 39]. Terms with both binomial and hypergeometric FDR *q*‐value < 0.05 were considered significant. Unlike traditional nearest‐gene approaches, GREAT associates regulatory regions with target genes by utilizing known regulatory domain definitions and accounts for the varying sizes of regulatory domains.

### 4.11 H3K27ac ChIP‐Seq

1 × 10^6 ^cells were harvested for three biological replicates of LPS‐treated BV2 cells under 1 and 23 kPa conditions. Pellets were crosslinked in 10 mL PBS with 1% FBS and 1% formaldehyde for 10 min at RT. Fixation was quenched with 200 mm glycine for 5 min at RT and 15 min on ice. Cells were then lysed using lysis buffer containing 1% SDS, 50 mm tris‐hcl pH 8, 10 mm EDTA, and protease inhibitor (Roche). Fixed chromatin was sonicated to a length of 300–400 bp using an M220 sonicator (Covaris). Sonicated chromatin was diluted 10‐fold with dilution buffer containing 0.1% Triton X‐100, 0.1% SDS, 150 mm NaCl, 15 mm Tris‐HCl pH 8, 1 mm EDTA, and protease inhibitor. Chromatin was incubated with Dynabeads Protein A (Invitrogen) and anti‐H3K27ac antibody (Active Motif, 39133) at 4°C overnight. Chromatin‐antibody‐bead complexes were thoroughly washed with RIPA wash buffer α containing 140 mm NaCl, 1 mm EDTA, 0.5 mm EGTA, 1% Triton X‐100, 0.1% SDS, 0.1% sodium deoxycholate, and 10 mm Tris‐HCl pH 8 to remove non‐specific complexes. RNA was removed from the complexes with RNase A (ThermoFisher) for 30 min at 37°C. Samples were reverse‐crosslinked with 0.3% SDS and 0.1 mg Proteinase K (NEB) at 65°C overnight. ChIP DNA was purified with AMPure XP beads (Beckman Coulter). ChIP‐seq libraries were generated using NEBNext Ultra II DNA Library Prep Kit (NEB) and sequenced with 100 bp paired‐end reads on the MGI DNBSEQ‐G400 platform. For ChIP‐seq data processing, paired‐end ChIP‐seq reads were aligned to the mouse reference genome mm10 using BWA‐MEM (ref: Aligning sequence reads, clone sequences and assembly contigs with BWA‐MEM). PCR duplicates were removed using Picard MarkDuplicates (ref: http://broadinstitute.github.io/picard/) and low‐quality reads (*MAPQ* < 10) were filtered out. H3K27ac peaks were identified using MACS2 [83] callpeak with a *q*‐value cutoff of 0.01. CREs were defined by merging H3K27ac peaks from all samples. To identify differential CREs, H3K27ac ChIP‐seq read counts on CREs were calculated using BEDTools coverage for each sample [84]. Differential testing between the 1 and 23 kPa conditions was performed using DESeq2 [85]. We defined rigidity‐upregulated differential CREs as mechanoCREs (*FDR* < 0.05), and unchanged CREs as static CREs (*FDR* ≥ 0.95). Reproducibility of mechanoCRE activities among replicates was measured by comparing scaled H3K27ac ChIP‐seq read counts obtained using the counts function of DESeq2. For downstream analysis and visualization, we merged the H3K27ac ChIP‐seq signals of replicates for 1 and 23 kPa conditions. For the tornado plots illustrating H3K27ac ChIP‐seq signals of mechanoCREs, deepTools bamCompare was used to calculate genome‐wide log_2_ (H3K27ac/input) signals, which were then plotted using deepTools plotHeatmap [86]. To visualize H3K27ac signals on genome tracks, we normalized H3K27ac ChIP‐seq signals using deepTools bamCoverage with CPM normalization [86].

### 4.12 In Situ Hi‐C

In situ Hi‐C experiments were performed as previously described [66] with a few modifications. 1 × 10^6 ^cells were harvested for two biological replicates of LPS‐treated BV2 cells under 1 and 23 kPa conditions. Pellets were crosslinked in 10 mL PBS with 1% FBS and 1% formaldehyde for 9 min at RT. Fixation was quenched with 200 mm glycine for 5 min at RT and 15 min on ice. Cells were then lysed with a lysis buffer containing 10 mm Tris‐HCl pH 8, 10 mm NaCl, and 0.2% IGEPAL CA630 for 15 min on ice. Nuclei were permeabilized using 0.5% SDS for 10 min at 62°C, and quenched with Triton X‐100 for 15 min at 37°C. Chromatin was digested with MboI (100 U; NEB) at 37°C for 2 h to digest chromatin, and deactivated for 20 min at 62°C. Digested chromatin was labelled with biotin‐14‐dCTP (Invitrogen) and 40 U of Klenow (NEB), and re‐ligated with 2000 U of T4 DNA Ligase (NEB) for 4 h at 23°C. Ligated samples were reverse‐crosslinked with 1 mg of Proteinase K (NEB), 1% SDS, and 500 mm NaCl at 68°C overnight. DNA was extracted using AMPure XP beads (Beckman Coulter) and sonicated to a length of 300–400 bp using an M220 sonicator (Covaris). Biotinylated DNA fragments were pulled down with Dynabeads MyOne streptavidin T1 beads (Invitrogen). To generate sequencing libraries, end repair was performed with 50 U of T4 PNK (NEB), 12 U of T4 DNA Polymerase (NEB), and 5 U of Klenow (NEB). Adenosine tails were added with 5 U of Klenow exo‐(NEB), and Illumina‐indexed adapters were ligated with 2 µL of DNA Quick Ligase (NEB). Finally, in situ Hi‐C libraries were amplified by PCR and sequenced with 100 bp paired‐end reads on the MGI DNBSEQ‐G400 platform. Hi‐C data were processed following the 4DN Hi‐C Processing Pipeline [87]. Briefly, sequenced paired‐end reads were mapped to the mouse reference genome mm10 using BWA‐MEM (ref: Aligning sequence reads, clone sequences and assembly contigs with BWA‐MEM). Mapped reads were sorted and filtered according to the pipeline criteria using Pairtools (10.5281/zenodo.1490830). Valid reads were converted into multi‐resolution (5 kb, 10 kb, 25 kb, 50 kb, 100 kb, 250 kb, 500 kb, 1 Mb, 2.5 Mb, 5 Mb, 10 Mb) contact matrices and stored in. hic format and. mcool format using Juicer and Cooler, respectively [88, 89]. The contact matrices were balanced using both the ICE and KR algorithms [66, 90]. Reproducibility of the Hi‐C data between replicates was measured by calculating stratum‐adjusted correlation coefficient values using HiCRep at a 10 kb resolution [91]. For downstream analysis and visualization, we merged the Hi‐C data of replicates for 1 and 23 kPa conditions.

To identify differential CREs, H3K27ac ChIP‐seq read counts on CREs were calculated using BEDTools coverage for each sample [84]. Differential testing between the 1 and 23 kPa conditions was performed using DESeq2 [85]. We defined differential CREs upon rigidity as mechanoCREs (*FDR* < 0.05). Static CREs were defined by excluding all CREs with *FDR* ≦ 0.95.

We categorized CREs as ‘promoter‐proximal’ if they were located within 500 bp of a known TSS, and as ‘promoter‐distal’ if they were not proximal but were significantly linked (ABC score > 0.02) to at least one known TSS. For promoter‐proximal CREs that overlapped multiple TSSs, the gene with the highest ABC score for each CRE was assigned as its target gene. The significant linkages between promoter‐distal CREs and TSSs were defined as CRE‐gene pairs. For mechanoCRE‐gene pairs, we selected upregulated and downregulated promoter‐distal mechanoCREs that were significantly linked to a TSS under the 23 and 1 kPa conditions, respectively. For static CRE‐gene pairs, we selected promoter‐distal static CREs significantly linked to the same TSS both in 1 and 23 kPa conditions.

To identify transcription factor motifs overrepresented in mechanoCREs relative to static CREs, de novo motif analysis was performed using the findMotifsGenome function of HOMER with the parameter ‘‐size given’ and static CREs as the background set [92]. Enriched motif candidates were selected only if they were included in the JASPAR database and had a similarity score above 0.7 [93].

To investigate A/B compartments in LPS‐treated BV2 cells under 1 and 23 kPa conditions, eigenvectors of the Hi‐C contact matrices for each replicate were calculated using the eigs‐*cis* function of Cooltools at a 100 kb resolution [94]. The eigenvectors from replicates of the same condition (1 and 23 kPa) were then averaged.

To analyze TAD boundaries, insulation scores and boundary strengths of the Hi‐C contact matrices for each replicate were calculated using the insulation function of Cooltools at a 10 kb resolution [94, 95]. Genomic regions were classified as TAD boundaries if they had a boundary strength greater than 0.5. TAD boundaries from replicates of the same condition were then merged. To compare TAD boundaries, two boundaries were considered overlapping if the distance between them was less than 20 kb, accounting for algorithmic inaccuracies in boundary calling.

For Hi‐C contact matrix and virtual 4C visualizations, we extracted KR‐normalized Hi‐C contact matrices using the dump function of Juicer at a 10 kb resolution [88]. We then performed quantile normalization on the KR‐normalized Hi‐C contact matrices from the 1 and 23 kPa conditions for each chromosome. For aggregated Hi‐C contact map analysis, the APA function of Juicer was applied at a 10 kb resolution to the KR‐normalized Hi‐C contact matrices with default parameters, targeting mechanoCRE‐gene pairs and static CRE‐gene pairs [88]. ‘NormedAPA’ data from the 1 and 23 kPa conditions were plotted [66].

### 4.13 Statistical Analysis

Statistical analyses were performed using GraphPad Prism software (version 9.5.1). Data normality was assessed using the Shapiro–Wilk test. For normally distributed data, statistical significance was evaluated using an unpaired two‐tailed Student's *t*‐test (for two‐group comparisons) or a one‐way ANOVA followed by Tukey's post‐hoc test (for comparisons of three or more groups). Significance thresholds were set at *p* < 0.05, *p* < 0.01, *p* < 0.001, and *p* < 0.0001. Data are presented as the mean ± standard deviation (SD) from three or four independent biological experiments. All fluorescence images were acquired using identical microscope settings (including exposure time, gain, and laser power), and representative images are shown in the figures. The specific sample sizes (*n*), representing the number of cells or regions of interest analyzed per condition, are detailed in the respective figure legends.

## Author Contributions

Y.‐X.M., J.J., Y.‐M.L., I.J., J.‐H.L., and H.‐W.K.: conceptualized the study and designed experiments; J.‐H.L. and H.‐W.K.: supervised the study; Y.‐X.M., C.‐J.L., S.‐J.S., B.‐B., J.‐H.L., and H.S.K.: characterized the samples and analyzed and validated the data. Y.‐X.M., J.J., T.‐W.H., S.‐L., J.‐C., B.‐E.Y., and J.‐H.L.: performed the in vitro cell and ex vivo studies. Y.‐X.M., I.J., and J.‐H.L.: drafted the manuscript; K.W.L., J.‐H.L., and H.‐W.K.: wrote and edited the manuscript.

## Conflicts of Interest

The authors declare no conflicts of interest.

## Supporting information




**Supporting File**: advs77736‐sup‐0001‐SuppMat.docx.

## Data Availability

The data that support the findings of this study are available from the corresponding author upon reasonable request.

## References

[1] D. Ricklin , G. Hajishengallis , K. Yang , and J. D. Lambris , “Complement: A Key System for Immune Surveillance and Homeostasis,” Nature Immunology 11, no. 9 (2010): 785–797, 10.1038/ni.1923. PMC2924908 20720586

[2] M. T. Heneka , M. P. Kummer , and E. Latz , “Innate Immune Activation in Neurodegenerative Disease,” Nature Reviews Immunology 14, no. 7 (2014): 463–477, 10.1038/nri3705. 24962261

[3] D. N. Xanthos and J. Sandkühler , “Neurogenic Neuroinflammation: Inflammatory CNS Reactions in Response to Neuronal Activity,” Nature Reviews Neuroscience 15, no. 1 (2014): 43–53, 10.1038/nrn3617. 24281245

[4] C. Gao , J. Jiang , Y. Tan , and S. Chen , “Microglia in Neurodegenerative Diseases: Mechanism and Potential Therapeutic Targets,” Signal Transduction and Targeted Therapy 8, no. 1 (2023): 1–37, 10.1038/s41392-023-01588-0. PMC10514343 37735487

[5] L. Wang , J. Xia , J. Li , et al., “Tissue and Cellular Rigidity and Mechanosensitive Signaling Activation in Alexander Disease,” Nature Communications 9, no. 1 (2018): 1899, 10.1038/s41467-018-04269-7. PMC5954157 29765022

[6] Y. Hu , G. Huang , J. Tian , et al., “Matrix Stiffness Changes Affect Astrocyte Phenotype in an In Vitro Injury Model,” NPG Asia Materials 13 (2021): 1–15.

[7] C. Wang , X. Tong , and F. Yang , “Bioengineered 3D Brain Tumor Model To Elucidate the Effects of Matrix Stiffness on Glioblastoma Cell Behavior Using PEG‐Based Hydrogels,” Molecular Pharmaceutics 11, no. 7 (2014): 2115–2125, 10.1021/mp5000828. 24712441

[8] Y. A. Miroshnikova , J. K. Mouw , J. M. Barnes , et al., “Tissue Mechanics Promote IDH1‐Dependent HIF1α–Tenascin C Feedback to Regulate Glioblastoma Aggression,” Nature Cell Biology 18, no. 12 (2016): 1336–1345, 10.1038/ncb3429. PMC5361403 27820599

[9] Y. A. Miroshnikova , J. K. Mouw , J. M. Barnes , et al., “Author Correction: Tissue Mechanics Promote IDH1‐Dependent HIF1α–Tenascin C Feedback to Regulate Glioblastoma Aggression,” Nature Cell Biology 25, no. 5 (2023): 787–788, 10.1038/s41556-023-01126-8. 37016139

[10] M. Imbault , C. Déméné , M. Mossad , et al., “Intraoperative Quantitative Measurement of Brain Tumor Stiffness and Intracranial Pressure Assessment Using Ultrasound Shear Wave Elastography,” in IEEE International Ultrasonics Symposium (IEEE, 2014): 201–204, https://ieeexplore.ieee.org/abstract/document/6932200.

[11] C. Walter , L. Crawford , M. Lai , et al., “Increased Tissue Stiffness in Tumors From Mice With Neurofibromatosis‐1 Optic Glioma,” Biophysical Journal 112, no. 8 (2017): 1535–1538, 10.1016/j.bpj.2017.03.017. PMC5406378 28445745

[12] A. Acharekar , K. Bachal , P. Shirke , et al., “Substrate Stiffness Regulates the Recurrent Glioblastoma Cell Morphology and Aggressiveness,” Matrix Biology 115 (2023): 107–127, 10.1016/j.matbio.2022.12.002. 36563706

[13] M. Cieśluk , K. Pogoda , P. Deptuła , et al., “Nanomechanics and Histopathology as Diagnostic Tools to Characterize Freshly Removed Human Brain Tumors,” International Journal of Nanomedicine 15 (2020): 7509–7521. 10.2147/IJN.S270147 PMC7547774 33116485

[14] T. J. Grundy , E. De Leon , K. R. Griffin , et al., “Differential Response of Patient‐Derived Primary Glioblastoma Cells to Environmental Stiffness,” Scientific Reports 6, no. 1 (2016): 23353, 10.1038/srep23353. PMC4800394 26996336

[15] S. Budday , R. Nay , R. de Rooij , et al., “Mechanical Properties of Gray and White Matter Brain Tissue by Indentation,” Journal of the Mechanical Behavior of Biomedical Materials 46 (2015): 318–330, 10.1016/j.jmbbm.2015.02.024. PMC4395547 25819199

[16] R. J. Henry , R. M. Ritzel , J. P. Barrett , et al., “Microglial Depletion With CSF1R Inhibitor During Chronic Phase of Experimental Traumatic Brain Injury Reduces Neurodegeneration and Neurological Deficits,” Journal of Neuroscience 40, no. 14 (2020): 2960–2974, 10.1523/JNEUROSCI.2402-19.2020. PMC7117897 32094203

[17] K. L. Monson , M. I. Converse , and G. T. Manley , “Cerebral Blood Vessel Damage in Traumatic Brain Injury,” Clinical Biomechanics 64 (2019): 98–113, 10.1016/j.clinbiomech.2018.02.011. 29478776

[18] J. Hu , Q. Chen , H. Zhu , et al., “Microglial Piezo1 Senses Aβ Fibril Stiffness to Restrict Alzheimer's Disease,” Neuron 111, no. 1 (2023): 15–29.e8, 10.1016/j.neuron.2022.10.021. 36368316

[19] F. Asci , M. Falletti , A. Zampogna , et al., “Rigidity in Parkinson's Disease: Evidence From Biomechanical and Neurophysiological Measures,” Brain 146 (2023): 3705–3718. 10.1093/brain/awad114 PMC10681667 37018058

[20] P. A. Netti , D. A. Berk , M. A. Swartz , A. J. Grodzinsky , and R. K. Jain , “Role of Extracellular Matrix Assembly in Interstitial Transport in Solid Tumors,” Cancer Research 60 (2000): 2497–2503. 10811131

[21] H. Du , J. M. Bartleson , S. Butenko , et al., “Tuning Immunity through Tissue Mechanotransduction,” Nature Reviews Immunology 23, no. 3 (2023): 174–188, 10.1038/s41577-022-00761-w. PMC9379893 35974148

[22] K. H. Vining , A. E. Marneth , K. Adu‐Berchie , et al., “Mechanical Checkpoint Regulates Monocyte Differentiation in Fibrotic Niches,” Nature Materials 21 (2022): 939–950. 10.1038/s41563-022-01293-3 PMC10197159 35817965

[23] S. W. Wong , S. Lenzini , M. H. Cooper , D. J. Mooney , and J.‐W. Shin , “Soft Extracellular Matrix Enhances Inflammatory Activation of Mesenchymal Stromal Cells to Induce Monocyte Production and Trafficking,” Science Advances 6, no. 15 (2020): aaw0158, 10.1126/sciadv.aaw0158. PMC7141831 32284989

[24] I. Andreu , I. Granero‐Moya , N. R. Chahare , et al., “Mechanical Force Application to the Nucleus Regulates Nucleocytoplasmic Transport,” Nature Cell Biology 24, no. 6 (2022): 896–905, 10.1038/s41556-022-00927-7. PMC7614780 35681009

[25] M. M. Nava , Y. A. Miroshnikova , L. C. Biggs , et al., “Heterochromatin‐Driven Nuclear Softening Protects the Genome Against Mechanical Stress‐Induced Damage,” Cell 181, no. 4 (2020): 800–817.e22, 10.1016/j.cell.2020.03.052. PMC7237863 32302590

[26] Y. Kalukula , A. D. Stephens , J. Lammerding , and S. Gabriele , “Mechanics and Functional Consequences of Nuclear Deformations,” Nature Reviews Molecular Cell Biology 23, no. 9 (2022): 583–602, 10.1038/s41580-022-00480-z. PMC9902167 35513718

[27] M. Reimer , S. Petrova Zustiak , S. Sheth , and J. M. Schober , “Intrinsic Response Towards Physiologic Stiffness Is Cell‐Type Dependent,” Cell Biochemistry and Biophysics 76, no. 1‐2 (2018): 197–208, 10.1007/s12013-017-0834-1. 29067585

[28] A. Momin , S. Bahrampour , H.‐K. Min , et al., “Channeling Force in the Brain: Mechanosensitive Ion Channels Choreograph Mechanics and Malignancies,” Trends in Pharmacological Sciences 42 (2021): 367–384. 10.1016/j.tips.2021.02.006 33752907

[29] Z. Xiang , M. Chen , J. Ping , et al., “Microglial Morphology and Its Transformation After Challenge by Extracellular ATP In Vitro,” Journal of Neuroscience Research 83, no. 1 (2006): 91–101, 10.1002/jnr.20709. 16323207

[30] J.‐K. Kim , S.‐B. Han , S. I. Park , I.‐S. Kim , and D.‐H. Kim , “Nuclear Transport of STAT6 Determines the Matrix Rigidity Dependent M2 Activation of Macrophages,” Biomaterials 290 (2022): 121859, 10.1016/j.biomaterials.2022.121859. 36306683

[31] A. Elosegui‐Artola , I. Andreu , A. E. M. Beedle , et al., “Force Triggers YAP Nuclear Entry by Regulating Transport Across Nuclear Pores,” Cell 171 (2017): 1397–1410. 10.1016/j.cell.2017.10.008 29107331

[32] S. Khetan , M. Guvendiren , W. R. Legant , D. M. Cohen , C. S. Chen , and J. A. Burdick , “Degradation‐Mediated Cellular Traction Directs Stem Cell Fate in Covalently Crosslinked Three‐Dimensional Hydrogels,” Nature Materials 12, no. 5 (2013): 458–465, 10.1038/nmat3586. PMC3633615 23524375

[33] C. J. Walker , C. Crocini , D. Ramirez , et al., “Nuclear Mechanosensing Drives Chromatin Remodelling in Persistently Activated Fibroblasts,” Nature Biomedical Engineering 5, no. 12 (2021): 1485–1499, 10.1038/s41551-021-00709-w. PMC9102466 33875841

[34] A. F. Lima , G. May , J. Díaz‐Colunga , et al., “Pires das Neves, Osmotic Modulation of Chromatin Impacts on Efficiency and Kinetics of Cell Fate Modulation,” Scientific Reports 8 (2018): 7210. 10.1038/s41598-018-25517-2 PMC5940679 29740078

[35] S. Heinz , C. E. Romanoski , C. Benner , and C. K. Glass , “The Selection and Function of Cell Type‐Specific Enhancers,” Nature Reviews Molecular Cell Biology 16, no. 3 (2015): 144–154, 10.1038/nrm3949. PMC4517609 25650801

[36] V. Venu , E. M. Small , C. Roth , et al., “Disruption of Histone Acetylation Homeostasis Reveals Multilayered Chromatin Regulation for Transcriptional Resiliency,” Epigenetics & Chromatin 18, no. 1 (2025): 63, 10.1186/s13072-025-00631-4. PMC12492967 41044756

[37] Y.‐M. Li , Y. Ji , Y.‐X. Meng , et al., “Neural Tissue‐Like, Not Supraphysiological, Electrical Conductivity Stimulates Neuronal Lineage Specification Through Calcium Signaling and Epigenetic Modification,” Advanced Science 11 (2024): 2400586. 10.1002/advs.202400586 PMC11425260 38984490

[38] C. Y. McLean , D. Bristor , M. Hiller , et al., “GREAT Improves Functional Interpretation of Cis‐Regulatory Regions,” Nature Biotechnology 28, no. 5 (2010): 495–501, 10.1038/nbt.1630. PMC4840234 20436461

[39] Y. Tanigawa , E. S. Dyer , and G. Bejerano , “WhichTF Is Functionally Important in Your Open Chromatin Data?,” PLOS Computational Biology 18, no. 8 (2022): 1010378, 10.1371/journal.pcbi.1010378. PMC9426921 36040971

[40] D. Kozono , J. Li , M. Nitta , et al., “Dynamic Epigenetic Regulation of Glioblastoma Tumorigenicity Through LSD1 Modulation of MYC Expression,” Proceedings of the National Academy of Sciences 112, no. 30 (2015): E4055–E4064, 10.1073/pnas.1501967112. PMC4522819 26159421

[41] B. L. Gregory and V. G. Cheung , “Natural Variation in the Histone Demethylase, KDM4C, Influences Expression Levels of Specific Genes Including Those That Affect Cell Growth,” Genome Research 24, no. 1 (2014): 52–63, 10.1101/gr.156141.113. PMC3875861 24285722

[42] M. J. Lathrop , The Role of Chromatin Regulation in Gene Expression: Analyzed Through the Phenotype Characterization of Chd6 ATPase‐/‐ Mice and Methylation Status of the Human Globin Locus, (PhD diss., Dartmouth College, 2009).

[43] D. P. Celias , K. Hänggi , and B. Ruffell , “Abstract 678: Investigating the Mechanisms Involved in HMGB1‐Dependent DNA Uptake and STING Activation in Dendritic Cells,” Cancer Research 83 (2023): 678.

[44] A. Casanova , S. H. Low , M. Québatte , et al., “A Role for the VPS Retromer in Brucella Intracellular Replication Revealed by Genomewide siRNA Screening,” mSphere 4, no. 3 (2019): 1–17, 10.1128/mSphere.00380-19. PMC6595151 31243080

[45] F. Liu , X. Cheng , C. Zhao , et al., “Single‐Cell Mapping of Brain Myeloid Cell Subsets Reveals Key Transcriptomic Changes Favoring Neuroplasticity After Ischemic Stroke,” Neuroscience Bulletin 40 (2023): 65–78, 10.1007/s12264-023-01109-7. PMC10774469 37755676

[46] T. Mukai , E. Di Martino , S. Tsuji , K. Blomgren , T. Nagamura‐Inoue , and U. Ådén , “Umbilical Cord‐Derived Mesenchymal Stromal Cells Immunomodulate and Restore Actin Dynamics and Phagocytosis of LPS‐Activated Microglia via PI3K/Akt/Rho GTPase Pathway,” Cell Death Discovery 7, no. 1 (2021): 1–13, 10.1038/s41420-021-00436-w. PMC7961004 33723246

[47] J. Karunia , A. Niaz , M. Mandwie , et al., “PACAP and VIP Modulate LPS‐Induced Microglial Activation and Trigger Distinct Phenotypic Changes in Murine BV2 Microglial Cells,” International Journal of Molecular Sciences 22, no. 20 (2021): 10947, 10.3390/ijms222010947. PMC8535941 34681607

[48] S. Jiao , C. Li , F. Guo , et al., “SUN1/2 Controls Macrophage Polarization via Modulating Nuclear Size and Stiffness,” Nature Communications 14, no. 1 (2023): 6416, 10.1038/s41467-023-42187-5. PMC10570371 37828059

[49] B. F. Olabiyi , A.‐C. Schmoele , E. C. Beins , and A. Zimmer , “Pharmacological Blockade of Cannabinoid Receptor 2 Signaling Does Not Affect LPS/IFN‐γ‐Induced Microglial Activation,” Scientific Reports 13, no. 1 (2023): 11105, 10.1038/s41598-023-37702-z. PMC10333177 37429837

[50] R. Medzhitov and T. Horng , “Transcriptional Control of the Inflammatory Response,” Nature Reviews Immunology 9, no. 10 (2009): 692–703, 10.1038/nri2634. 19859064

[51] A. Roy , M. Srivastava , U. Saqib , et al., “Potential Therapeutic Targets for Inflammation in Toll‐Like Receptor 4 (TLR4)‐Mediated Signaling Pathways,” International Immunopharmacology 40 (2016): 79–89, 10.1016/j.intimp.2016.08.026. 27584057

[52] J. Hey , M. Paulsen , R. Toth , et al., “Epigenetic Reprogramming of Airway Macrophages Promotes Polarization and Inflammation in Muco‐Obstructive Lung Disease,” Nature Communications 12, no. 1 (2021): 6520, 10.1038/s41467-021-26777-9. PMC8586227 34764283

[53] L. Yu , X. Weng , P. Liang , et al., “MRTF‐A Mediates LPS‐Induced Pro‐Inflammatory Transcription by Interacting With the COMPASS Complex,” Journal of Cell Science 127 (2014): 4645–4657. 10.1242/jcs.152314 25189621

[54] X. Shiwen , R. Stratton , J. Nikitorowicz‐Buniak , et al., “A Role of Myocardin Related Transcription Factor‐A (MRTF‐A) in Scleroderma Related Fibrosis,” PLoS One 10, no. 5 (2015): 0126015, 10.1371/journal.pone.0126015. PMC4425676 25955164

[55] H. Xu , K. Wei , J. Ni , et al., “Matrix Stiffness Regulates Nucleus Pulposus Cell Glycolysis by MRTF‐A‐Dependent Mechanotransduction,” Bone Research 13, no. 1 (2025): 23, 10.1038/s41413-025-00402-7. PMC11828926 39952914

[56] L. Yang , J. Li , G. Zang , et al., “Pin1/ YAP Pathway Mediates Matrix Stiffness‐Induced Epithelial–Mesenchymal Transition Driving Cervical Cancer Metastasis via a Non‐Hippo Mechanism,” Bioengineering & Translational Medicine 8, no. 1 (2023): 10375, 10.1002/btm2.10375. PMC9842039 36684109

[57] N. Ronkina , J. Lafera , A. Kotlyarov , and M. Gaestel , “Stress‐Dependent Phosphorylation of Myocardin‐Related Transcription Factor A (MRTF‐A) by the p38MAPK/MK2 Axis,” Scientific Reports 6, no. 1 (2016): 31219, 10.1038/srep31219. PMC4974569 27492266

[58] S. Dupont , L. Morsut , M. Aragona , et al., “Role of YAP/TAZ in Mechanotransduction,” Nature 474 (2011): 179–183, https://www.nature.com/articles/nature10137. 10.1038/nature10137 21654799

[59] P.‐S. Liu , Y.‐T. Chen , X. Li , et al., “CD40 Signal Rewires Fatty Acid and Glutamine Metabolism for Stimulating Macrophage Anti‐Tumorigenic Functions,” Nature Immunology 24, no. 3 (2023): 452–462, 10.1038/s41590-023-01430-3. PMC9977680 36823405

[60] R. Li , M. Meng , Y. Chen , et al., “ATP‐Citrate Lyase Controls Endothelial Gluco‐Lipogenic Metabolism and Vascular Inflammation in Sepsis‐Associated Organ Injury,” Cell Death & Disease 14 (2023): 1–14. 10.1038/s41419-023-05932-8 PMC10325983 37414769

[61] Y.‐H. Gao , Y. Zhang , Y.‐X. Guo , et al., “Treatment With Anacardic Acid Modulates Dendritic Cell Activation and Alleviates the Disease Development of Autoimmune Neuroinflammation in Mice,” Biochemical and Biophysical Research Communications 613 (2022): 34–40, 10.1016/j.bbrc.2022.04.115. 35526486

[62] C.‐H. Yang , L. Fagnocchi , S. Apostle , et al., “Independent Phenotypic Plasticity Axes Define Distinct Obesity Sub‐Types,” Nature Metabolism 4, no. 9 (2022): 1150–1165, 10.1038/s42255-022-00629-2. PMC9499872 36097183

[63] E. Acaz‐Fonseca , A. Ortiz‐Rodriguez , I. Azcoitia , L. M. Garcia‐Segura , and M.‐A. Arevalo , “Notch Signaling in Astrocytes Mediates Their Morphological Response to an Inflammatory Challenge,” Cell Death Discovery 5, no. 1 (2019): 1–14, 10.1038/s41420-019-0166-6. PMC6447583 30962951

[64] E. Lieberman‐Aiden , N. L. van Berkum , L. Williams , et al., “Comprehensive Mapping of Long‐Range Interactions Reveals Folding Principles of the Human Genome,” Science 326, no. 5950 (2009): 289–293, 10.1126/science.1181369. PMC2858594 19815776

[65] J. R. Dixon , S. Selvaraj , F. Yue , et al., “Topological Domains in Mammalian Genomes Identified by Analysis of Chromatin Interactions,” Nature 485, no. 7398 (2012): 376–380, 10.1038/nature11082. PMC3356448 22495300

[66] S. S. P. Rao , M. H. Huntley , N. C. Durand , et al., “A 3D Map of the Human Genome at Kilobase Resolution Reveals Principles of Chromatin Looping,” Cell 159, no. 7 (2014): 1665–1680, 10.1016/j.cell.2014.11.021. PMC5635824 25497547

[67] J. R. Dixon , I. Jung , S. Selvaraj , et al., “Chromatin Architecture Reorganization During Stem Cell Differentiation,” Nature 518, no. 7539 (2015): 331–336, 10.1038/nature14222. PMC4515363 25693564

[68] B. Mifsud , F. Tavares‐Cadete , A. N. Young , et al., “Mapping Long‐range Promoter Contacts in Human Cells With High‐Resolution Capture Hi‐C,” Nature Genetics 47, no. 6 (2015): 598–606, 10.1038/ng.3286. 25938943

[69] I. Jung , A. Schmitt , Y. Diao , et al., “A Compendium of Promoter‐Centered Long‐Range Chromatin Interactions in the Human Genome,” Nature Genetics 51, no. 10 (2019): 1442–1449, 10.1038/s41588-019-0494-8. PMC6778519 31501517

[70] K. P. McCreery , A. Stubb , R. Stephens , et al., “Mechano‐Osmotic Signals Control Chromatin State and Fate Transitions in Pluripotent Stem Cells,” Nature Cell Biology 27, no. 10 (2025): 1757–1770, 10.1038/s41556-025-01767-x. PMC12527910 41023488

[71] C. P. Fulco , J. Nasser , and T. R. Jones , et al., “Activity‐by‐contact Model of Enhancer–promoter Regulation From Thousands of CRISPR Perturbations,” Nature Genetics 51, no. 12 (2019): 1664–1669, 10.1038/s41588-019-0538-0. PMC6886585 31784727

[72] M. Li , Y.‐M. Kim , J. H. Koh , et al., “Serum Amyloid A Expression in Liver Promotes Synovial Macrophage Activation and Chronic Arthritis via NFAT5,” Journal of Clinical Investigation 134 (2024): 167835. 10.1172/JCI167835 PMC10904059 38426494

[73] P. Tzerpos , D. Bojcsuk , K. Bene , et al., “The Transcription Factor BACH1 Couples Chromatin Priming and Repression to Enable Macrophage Plasticity and Adaptation,” Immunity 59 (2026): 2465–2482.e8. 10.1016/j.immuni.2026.06.022 42468527

[74] B. Xu , C. Tang , R. Han , et al., “Targeting the Chemokine‐Microglia Nexus: A Novel Strategy for Modulating Neuroinflammation in Alzheimer's Disease,” Journal of Alzheimer's Disease Reports 9 (2025): 25424823251326044, 10.1177/25424823251326044. PMC12049630 40321241

[75] Y. Jin , Y. Kang , M. Wang , et al., “Targeting Polarized Phenotype of Microglia via IL6/JAK2/STAT3 Signaling to Reduce NSCLC Brain Metastasis,” Signal Transduction and Targeted Therapy 7, no. 1 (2022): 52, 10.1038/s41392-022-00872-9. PMC8864012 35194016

[76] J. Groh , R. Feng , X. Yuan , et al., “Microglia Activation Orchestrates CXCL10‐Mediated CD8+ T Cell Recruitment to Promote Aging‐Related White Matter Degeneration,” Nature Neuroscience 28 (2025): 1160–1173, https://www.nature.com/articles/s41593‐025‐01955‐w. 10.1038/s41593-025-01955-w PMC12148934 40404995

[77] O. Butovsky , M. P. Jedrychowski , C. S. Moore , et al., “Identification of a Unique TGF‐β–Dependent Molecular and Functional Signature in Microglia,” Nature Neuroscience 17, no. 1 (2014): 131–143, 10.1038/nn.3599. PMC4066672 24316888

[78] M. Metzemaekers , A. Mortier , A. Vacchini , et al., “Endogenous Modification of the Chemoattractant CXCL5 Alters Receptor Usage and Enhances Its Activity Toward Neutrophils and Monocytes,” Science Signaling 14, no. 673 (2021): aax3053, 10.1126/scisignal.aax3053. 33688078

[79] Y. Wang , W. Wang , L. Su , et al., “BACH1 Changes Microglial Metabolism and Affects Astrogenesis During Mouse Brain Development,” Developmental Cell 59 (2024): 108–124.e7, https://www.cell.com/developmental‐cell/fulltext/S1534‐5807(23)00616‐0. 10.1016/j.devcel.2023.11.018 38101413

[80] J. R. Tse and A. J. Engler , “Preparation of Hydrogel Substrates With Tunable Mechanical Properties,” Current Protocols Cell Biology 47 (2010): 10.16.1–10.16.16,. 10.1002/0471143030.cb1016s47 20521229

[81] H. Lian , E. Roy , and H. Zheng , “Protocol for Primary Microglial Culture Preparation,” Bio‐Protocol 6, no. 21 (2016): 1989, 10.21769/BioProtoc.1989. PMC5669279 29104890

[82] J. D. Buenrostro , B. Wu , H. Y. Chang , and W. J. Greenleaf , “ATAC‐Seq: A Method for Assaying Chromatin Accessibility Genome‐Wide,” Current Protocols in Molecular Biology 109, no. 1 (2015): 21.29.1–21.29.9, 10.1002/0471142727.mb2129s109. PMC4374986 25559105

[83] Y. Zhang , T. Liu , C. A. Meyer , et al., “Model‐Based Analysis of ChIP‐Seq (MACS),” Genome Biology 9, no. 9 (2008): R137, 10.1186/gb-2008-9-9-r137. PMC2592715 18798982

[84] A. R. Quinlan and I. M. Hall , “BEDTools: A Flexible Suite of Utilities for Comparing Genomic Features,” Bioinformatics 26, no. 6 (2010): 841–842, 10.1093/bioinformatics/btq033. PMC2832824 20110278

[85] M. I. Love , W. Huber , and S. Anders , “Moderated Estimation of Fold Change and Dispersion for RNA‐Seq Data With DESeq2,” Genome Biology 15, no. 12 (2014): 550, 10.1186/s13059-014-0550-8. PMC4302049 25516281

[86] F. Ramírez , D. P. Ryan , B. Grüning , et al., “deepTools2: A Next Generation Web Server for Deep‐Sequencing Data Analysis,” Nucleic Acids Research 44 (2016): W160–165. 10.1093/nar/gkw257 PMC4987876 27079975

[87] S. B. Reiff , A. J. Schroeder , K. Kırlı , et al., “The 4D Nucleome Data Portal as a Resource for Searching and Visualizing Curated Nucleomics Data,” Nature Communications 13, no. 1 (2022): 2365, 10.1038/s41467-022-29697-4. PMC9061818 35501320

[88] N. C. Durand , M. S. Shamim , I. Machol , et al., “Juicer Provides a One‐Click System for Analyzing Loop‐Resolution Hi‐C Experiments,” Cell Systems 3, no. 1 (2016): 95–98, 10.1016/j.cels.2016.07.002. PMC5846465 27467249

[89] N. Abdennur and L. A. Mirny , “Cooler: Scalable Storage for Hi‐C Data and Other Genomically Labeled Arrays,” Bioinformatics 36, no. 1 (2020): 311–316, 10.1093/bioinformatics/btz540. PMC8205516 31290943

[90] M. Imakaev , G. Fudenberg , R. P. McCord , et al., “Iterative Correction of Hi‐C Data Reveals Hallmarks of Chromosome Organization,” Nature Methods 9, no. 10 (2012): 999–1003, 10.1038/nmeth.2148. PMC3816492 22941365

[91] T. Yang , F. Zhang , G. G. Yardımcı , et al., “HiCRep: assessing the Reproducibility of Hi‐C Data Using a Stratum‐adjusted Correlation Coefficient,” Genome Research 27, no. 11 (2017): 1939–1949, 10.1101/gr.220640.117. PMC5668950 28855260

[92] S. Heinz , C. Benner , N. Spann , et al., “Simple Combinations of Lineage‐Determining Transcription Factors Prime Cis‐Regulatory Elements Required for Macrophage and B Cell Identities,” Molecular Cell 38, no. 4 (2010): 576–589, 10.1016/j.molcel.2010.05.004. PMC2898526 20513432

[93] I. Rauluseviciute , R. Riudavets‐Puig , R. Blanc‐Mathieu , et al., “JASPAR 2024: 20th Anniversary of the Open‐Access Database of Transcription Factor Binding Profiles,” Nucleic Acids Research 52, no. D1 (2024): D174–D182, 10.1093/nar/gkad1059. PMC10767809 37962376

[94] Open2C , N. Abdennur , S. Abraham , G. Fudenberg , et al., “Cooltools: Enabling High‐Resolution Hi‐C Analysis in Python,” PLOS Computational Biology 20, no. 5 (2024): 1012067, 10.1371/journal.pcbi.1012067. PMC11098495 38709825

[95] E. Crane , Q. Bian , R. P. McCord , et al., “Condensin‐Driven Remodelling of X Chromosome Topology During Dosage Compensation,” Nature 523, no. 7559 (2015): 240–244, 10.1038/nature14450. PMC4498965 26030525

