# Supplementary material for: Matrix Rigidity Mechanoprimes Microglia for Inflammation Through Cytoskeletal‐to‐Nuclear Signaling and 3D Spatio‐Epigenomic Remodeling
Source: Adv Sci (Weinh). 2026 Sep 27:e77736. Online ahead of print. doi: 10.1002/advs.77736 (PMC13616372; doi:10.1002/advs.77736)
Supplement: Supplementary file 1 [file ADVS-9999-e77736-s001.docx]

**Supplementary Figures and Tables**

- Fig. S1-S24

- Table S1-S5

**Fig. S1. Microglial morphological adaptations to matrix rigidity.** (a) Representative optical microscopy images of microglial (BV2) cells cultured on fibronectin-coated polyacrylamide (PAA) hydrogels of varying stiffness (1, 7, 10, 23, and 100 kPa) at low and high magnifications. (b) Low-magnification confocal images of microglia cultured on compliant (1 kPa) and rigid (23 kPa) matrices for 12 h. Corresponding high-magnification images are provided in Fig. 2c. (c) Representative confocal z-stack images detailing the F-actin cytoskeleton (phalloidin) and nuclear morphology (DAPI) of microglia on compliant and rigid matrices. Cross-sectional (XY) planes are displayed at the apical, equatorial, and basal levels. Enlarged apical-plane views highlight pronounced F-actin ruffling on rigid substrates.

**Fig. S2. Schematic representation of nuclear morphological analysis.** The diagram illustrates the parameters measured to assess nuclear deformation on compliant (1 kPa) versus rigid (23 kPa) matrices, including the 2D nuclear projection area, 3D nuclear volume, and the nuclear flattening index (defined as the ratio of the longest nuclear axis to nuclear height).

**Fig. S3. Primary microglia adapt morphology and nuclear shape to matrix rigidity.** **(a)** Representative confocal images of primary microglia, isolated from neonatal mice, cultured on compliant (1 kPa) and rigid (23 kPa) substrates. Staining for F-actin (green) and nuclei (DAPI, blue) highlights morphological changes in response to matrix stiffness. **(b)** Quantification of 2D projected cell area (*n* = 47) and F-actin intensity (*n* = 53). **(c)** Characterization of nuclear deformation, including 2D nuclear area (*n* = 50), height (*n* = 51), flattening index (*n* = 53), and 3D volume (*n* = 52). Statistical significance was determined via Student’s t-test: *****P* < 0.0001, ***P* < 0.01.

**Fig. S4. Matrix rigidity enlarges nuclear pores and enhances molecular nuclear transport.** **(a)** Transmission electron microscopy (TEM) analysis after 12 h of culture reveals a significant enlargement (~20%) of nuclear pores on rigid (23 kPa) compared to compliant (1 kPa) matrices (90.5 nm vs. 75.4 nm). Red brackets indicate the nuclear pore regions. **(b)** Real-time fluorescence imaging of 70 kDa dextran, utilized as a model macromolecule, demonstrating its transport into the nucleus. Statistical significance was determined via Student’s t-test: ***P* < 0.01.

**Fig. S5. Methacrylated hyaluronic acid (MeHA) hydrogels provide a dynamically stiffening cell culture platform.** MeHA hydrogels were functionalized with RGD peptide sequences to facilitate cell adhesion. UV exposure enabled the controlled modulation of gel stiffness from a compliant (1 kPa) to a rigid (20 kPa) state, allowing for precise temporal regulation of the mechanical microenvironment.

**Fig. S6. Matrix rigidity induces nuclear decondensation and increases histone acetylation in primary microglia.** **(a, b)** Analysis of chromatin condensation. **(a)** Representative images of DAPI-stained nuclei displayed with a pseudocolor (rainbow) intensity map, and **(b)** corresponding quantification of chromatin condensation, defined as the coefficient of variation (SD/mean) intensity of DAPI staining) (*n* = 50). **(c, d)** Analysis of histone acetylation. **(c)** Representative immunofluorescence images of acetylated histone H3 (H3ace) and **(d)** quantification of average H3ace fluorescence intensity, normalized to the 1 kPa condition (*n* = 40). Sample sizes (*n*) represent the number of cells or regions of interest (ROIs) analyzed per condition. Statistical significance was determined via Student’s t-test: **** *P* < 0.0001, *** *P* < 0.001.

**Fig. S7. Super-resolution N-SIM analysis of nuclear architecture.** Line-scan analysis of H3ace (green) and DNA (DAPI, blue) fluorescence intensities. The graphs display normalized fluorescence intensity profiles for three representative cells, measured along the indicated red dotted arrows.

**Fig. S8. Quality control and validation of ATAC-seq in microglia cultured on compliant and rigid matrices.** **(a)** Confirmation of the absence of mycoplasma contamination in microglial (BV2) cell cultures. **(b–e)** ATAC-seq validation and analysis of open chromatin regions in cells cultured on compliant (1 kPa) and rigid (23 kPa) matrices. **(b)** Pie charts detailing the genomic distribution of accessible regions across functional genomic features, including introns, exons, intergenic regions, 3’ UTRs, 5’ UTRs, promoters/transcription start sites (TSS), transcription end sites (TES), and non-coding regions. Promoter-TSS regions are defined as peak summits located up to 1 kb upstream and 100 bp downstream of the TSS. **(c)** Heatmaps illustrating read enrichment at the TSS for the 1 kPa and 23 kPa ATAC-seq libraries. The observed enrichment patterns align with characteristic ATAC-seq profiles, validating the high quality of the sequencing results. **(d)** Total number of chromatin accessibility peaks called for each experimental group. **(e)** Summary of key quality control metrics for the ATAC-seq experiments. Data are derived from two independent biological replicates per condition.

**Fig. S9. Genomic distribution of C1 and C2 DARs.** (left) Stacked bar representation of the genomic annotation of C1 (n = 1,729) and C2 (n = 2,567) DARs. (right) Feature-wise comparison between clusters. Statistical comparison was performed by two-sided Fisher's exact test for each genomic feature, with Benjamini–Hochberg correction for multiple testing across the seven features. *****q* < 0.0001. C1 DARs were significantly enriched at promoters (odds ratio = 1.85, *q* = 3.4×10⁻¹⁴), whereas C2 DARs were enriched in distal intergenic regions (odds ratio = 0.66, *q* = 2.2×10⁻¹⁰). All other genomic features showed no significant difference between clusters.

**Fig. S10. Gene Ontology (GO) analysis of biological processes associated with rigidity-induced DARs.** Functional enrichment analysis of differentially accessible regions (DARs) identified via ATAC-seq comparing rigid (23 kPa) to compliant (1 kPa) matrices. The results reveal a significant enrichment of key biological processes, including mechanotransduction and inflammation, as determined by Genomic Regions Enrichment of Annotations Tool (GREAT) analysis.

**Fig. S11. GO molecular function and KEGG pathway analyses of rigidity-induced DARs.** Functional enrichment analyses of differentially accessible regions (DARs) identified via ATAC-seq comparing rigid (23 kPa) to compliant (1 kPa) matrices. The results reveal a significant enrichment of molecular functions and signaling pathways associated with chromatin opening, mechanotransduction, and inflammation, as determined by **(a)** nearest gene analysis and **(b)** Genomic Regions Enrichment of Annotations Tool (GREAT) analysis.

**Fig. S12. Rigidity-induced DARs are predominantly enriched in non-exonic regulatory regions.** Genomic distribution analysis of differentially accessible regions (DARs) reveals a significant enrichment (>94%) within non-exonic regions, particularly distal intergenic elements. This profound enrichment indicates that extensive non-coding regulatory mechanisms underlie the mechanosensitive activation of microglia. DARs were defined using a fold change ≥ 1.5, *P* < 0.05, and a normalized read count > 2 (*n* = 2 biological replicates).

**Fig. S13. ATAC-seq signal tracks for representative genes associated with chromatin opening, cytoskeletal regulation, and inflammation.** Tracks display chromatin accessibility at representative loci for chromatin opening (*Chd6*, *Kdm4c*, *Hmgb1*), cytoskeletal regulation (*Amot*), and inflammation (*Ikbke*, *Stat1*). In all tracks, non-coding differentially accessible regions (DARs) are magnified to highlight regulatory changes. The y-axis represents peak signal intensity, and the x-axis indicates genomic position across the respective gene loci.

**Fig. S14. *De novo* motif analysis of rigidity-induced transcription factor binding sites.** Microglial cells cultured on compliant (1 kPa) and rigid (23 kPa) matrices were subjected to ATAC-seq. Subsequent *de novo* motif analysis of the resulting differentially accessible regions was performed to identify transcription factor binding motifs enriched in response to matrix rigidity.

**Fig. S15. Proposed mechanism of rigidity-driven microglial mechanopriming and cooperative inflammatory activation.** Matrix rigidity induces F-actin cytoskeletal remodeling and subsequent nuclear deformation in microglia. These mechanoadaptations alter the chromatin landscape, establishing a "mechanoprimed" state characterized by increased chromatin accessibility at inflammatory genomic loci. Upon exposure to secondary biochemical stimuli, the expression of inflammatory genes is synergistically amplified. This amplification is facilitated by the mechanosensitive transcriptional coactivator MRTF-A, which translocates to the nucleus under rigid matrix conditions. The schematic illustrates the hypothesized interplay wherein MRTF-A cooperates with biochemical inflammatory regulators at these newly accessible loci, culminating in the robust coactivation of inflammatory genes. Rigidity-driven mechanopriming and activation events are highlighted in red, whereas standard biochemical signaling pathways are depicted in gray. The proposed synergistic complex formation at the genomic loci is rendered in muted colors.

**Fig. S16. Combined effects of matrix rigidity and LPS treatment on microglial morphology.** **(a)** Experimental timeline for lipopolysaccharide (LPS) stimulation. Microglial (BV2) cells were cultured on compliant (1 kPa) and rigid (23 kPa) PAA gels for 12 h, followed by LPS treatment for an additional 12 h. **(b)** Representative low-magnification fluorescence images of cells stained for F-actin (phalloidin). **(c)** Quantitative morphological analysis derived from F-actin imaging, detailing cell roundness, perimeter, F-actin intensity, and 2D projected cell area. F-actin fluorescence intensity was normalized to the unstimulated 1 kPa control condition.

**Fig. S17. Matrix rigidity and LPS stimulation synergistically enhance microglial inflammatory activation.** **(a)** Quantitative morphological analysis derived from F-actin imaging, detailing cell roundness, perimeter, F-actin intensity, and 2D projected cell area. F-actin fluorescence intensity was normalized to the unstimulated 1 kPa control condition. **(b)** Western blot analysis of iNOS expression, with relative protein levels normalized to $\beta$-actin indicated by the italicized values below each lane. **(c, d)** Immunofluorescence analysis of the microglial activation marker Iba1. **(c)** Representative confocal images of microglia immunostained for Iba1 (red), F-actin (phalloidin, green), and nuclei (DAPI, blue). **(d)** Semi-quantification of average Iba1 fluorescence intensity.

**Fig. S18. Actin cytoskeletal disruption reverses rigidity-induced inflammatory transcriptomic profiles in microglia.** Microglial (BV2) cells were cultured for 12 h on compliant (1 kPa) or rigid (23 kPa) substrates. A subset of cells on 23 kPa matrices was pretreated with 1.5 μM cytochalasin D (CytoD) for the final 1 h of culture. All groups were subsequently stimulated with lipopolysaccharide (LPS) prior to RNA-seq analysis. **(a)** Principal component analysis (PCA) demonstrating distinct clustering based on substrate rigidity and cytoskeletal integrity. Notably, CytoD treatment shifts the transcriptomic profile of rigid-substrate samples closer to that of compliant-substrate samples. **(b)** Venn diagram identifying 215 inversely regulated genes shared between the rigidity comparison (23 kPa vs. 1 kPa) and the cytoskeletal disruption comparison (23 kPa + CytoD vs. 23 kPa). **(c)** Gene Ontology (GO) bubble plot illustrating enriched biological processes (BP) and cellular components (CC), highlighting inflammation-associated (red) and mechanotransduction-related (blue) pathways. Bubble size corresponds to the gene count, and color intensity reflects statistical significance (*P*-value).

**Fig. S19. Matrix rigidity enhances** SRF expression and **requires** serum **to amplify** inflammatory responses. **(a)** Representative immunofluorescence images of serum response factor (SRF) expression in microglia cultured on compliant (1 kPa) and rigid (23 kPa) substrates with LPS treatment. Cells were co-stained for F-actin (green), SRF (red), and nuclei (DAPI, blue). Quantification reveals a significantly higher total SRF intensity and nuclear-to-cytoplasmic (N/C) ratio on 23 kPa. Scale bar = 50 µm. **(b)** Western blot analysis confirms elevated SRF levels on 23 kPa versus 1 kPa under LPS stimulation, which are maintained even with CytoD treatment. **(c)** Analysis of iNOS expression in serum-free medium shows no significant difference between 1 kPa and 23 kPa with LPS, demonstrating that serum-dependent SRF signaling is required for rigidity-mediated inflammatory amplification. (d) Immunofluorescence images of LPS-stimulated microglia stained for DAPI (blue), F-actin (phalloidin, green), and YAP (red) on 1 kPa and 23 kPa matrices, with quantification of the YAP nuclear-to-cytoplasmic ratio (n = 90 cells per condition). Scale bar = 50 µm. Microglial (BV2) cells were cultured for 12 h before LPS stimulation (1 µg/mL for an additional 12 h). Statistical significance was determined using Student’s t-test: **P* < 0.05, *****P* < 0.0001.

**Fig. S20. Matrix rigidity reduces chromatin condensation and increases H3 acetylation in LPS-treated primary microglia.** **(a)** Representative images of DAPI-stained nuclei, displayed using a pseudocolor (rainbow) lookup table, and **(b)** quantification of chromatin condensation, calculated as the coefficient of variation (SD/mean) of DAPI fluorescence intensity (*n* = 48). **(c)** Representative immunofluorescence images of acetylated histone H3 (H3ace) (*n* = 34), and **(d**) semi-quantification of H3ace fluorescence intensity, normalized to the compliant (1 kPa) condition. Sample sizes (*n*) represent the number of cells or ROIs analyzed per condition. Statistical significance was determined using Student’s *t*-test: ****P* < 0.001, **P* < 0.05.

**Fig. S21. Pharmacological inhibition of histone acetyltransferases (HATs) using C646 or anacardic acid (AA) reverses rigidity-induced chromatin decondensation.** Microglial (BV2) cells were cultured on compliant (1 kPa) or rigid (23 kPa) matrices and pretreated with C646 (20 μM) or AA (50 μM) for 1 h, followed by 12 h of LPS treatment. **(a)** Western blot analysis confirming reduced acetylated histone H3 (H3ace) levels following HAT inhibition. **(b)** Representative confocal microscopy images demonstrating the reduced nuclear accumulation of H3ace (red) in BV2 microglia pretreated with HAT inhibitors. Nuclei were counterstained with DAPI (blue). **(c)** High-resolution representative images of DAPI-stained nuclei on rigid matrices. Treatments with C646 or AA counteracted rigidity-induced nuclear decondensation similarly to CytoD. **(d)** Semi-quantitative analysis of chromatin condensation (visualized via the rainbow intensity scale in c) confirms these findings. Statistical significance was assessed using one-way ANOVA followed by Tukey’s post-hoc test; significance levels are indicated in the respective graphs.

**Fig. S22. HAT inhibitors (C646 and AA) suppress inflammatory and HAT gene expression induced by matrix rigidity.** Quantitative real-time PCR (RT-qPCR) analysis demonstrating the rigidity-induced upregulation of inflammatory genes (*iNOS*, *IL-6*) and HAT genes (*Ep300*, *Crebbp*) in microglial (BV2) cells. This upregulation on rigid (23 kPa) versus compliant matrices (1 kPa) is effectively reversed by disrupting the actin cytoskeleton with CytoD or by treatment with the pharmacological HAT inhibitors C646 and AA. Data are presented as mean ± SD from three independent biological experiments. Statistical significance was determined using one-way ANOVA followed by Tukey's post-hoc test: *****P* < 0.0001, ****P* < 0.001, ***P* < 0.01; ns, not significant (*P* > 0.05).

**Fig. S23. Rigidity-induced microglial activation exacerbates astrocyte reactivity and compromises cell viability.** **(a)** Experimental setup illustrating the treatment of primary astrocytes with conditioned medium from microglia cultured on compliant (1 kPa) or rigid (23 kPa) matrices with LPS stimulation, followed by RT-qPCR analysis, morphological examination, and cell viability assessment. **(b, c)** Representative immunofluorescence images of astrocytes (immunostained for GFAP, red; nuclei, DAPI, blue) showing morphological changes (polygonal, bipolar, or stellate) indicative of reactive astrocytes when treated with microglia-conditioned medium. **(d)** Heatmap displaying the expression of inflammatory genes (*IL-6* and *TNF-α*) analyzed by RT-qPCR and the impact on astrocyte viability induced by microglia-conditioned medium. **(e)** Quantitative assessment of astrocyte cell viability following treatment with conditioned medium from microglia cultured under compliant or rigid matrix conditions with LPS. Data are presented as mean ± SD from three independent biological replicates. Statistical significance was assessed using one-way ANOVA followed by Tukey’s post-hoc test: *****P* < 0.0001, ****P* < 0.001, ***P* < 0.01.

**Fig. S24. Analysis of H3K27ac ChIP-seq and *in situ* Hi-C data. (a)** Bar plots showing the reproducibility of *in situ* Hi-C data between biological replicates (*n* = 2). Stratum-adjusted correlation coefficient (SCC) values between the biological replicates are displayed. **(b)** Conservation of higher-order chromatin structures. Exemplified Hi-C contact maps of LPS-treated BV2 cells under 1 kPa (lower diagonal) and 23 kPa (upper diagonal) conditions. The PC1 eigenvector values and insulation scores of LPS-treated BV2 cells under 1 kPa (bottom) and 23 kPa (right) conditions are shown, representing compartment A/B and TAD structures. **(c)** Scatter plot comparing genome-wide PC1 eigenvector values between LPS-treated BV2 cells under 1 kPa (*x*-axis) and 23 kPa (*y*-axis) conditions. **(d)** Venn diagram comparing TAD boundaries between LPS-treated BV2 cells under 1 kPa (blue) and 23 kPa (red) conditions. **(e)** Heatmap illustrating the reproducibility of H3K27ac ChIP-seq signals among biological replicates (n = 3) of mechanoCREs. Colors represent normalized H3K27ac ChIP-seq read counts. **(f)** Pie charts depicting the percentages of promoter-distal, promoter-proximal, and unclassified CREs within all CREs obtained from 1 kPa (top) and 23 kPa (bottom) under LPS treatment. **(g)** Transcription factor motifs overrepresented in upregulated relative to downregulated (top) and in downregulated relative to upregulated (bottom) promoter-distal mechanoCREs are ranked by enrichment. The top five enriched motifs are highlighted, and motifs corresponding to immune-related regulators are highlighted in red. **(h)** Transcription factor motifs overrepresented in upregulated (top) and downregulated (bottom) promoter-proximal mechanoCREs are ranked by enrichment. The top five enriched motifs are highlighted, and motifs corresponding to immune-related regulators are highlighted in red. The y-axis scale corresponds to that used for each corresponding type of mechanoCRE in Fig. 7c. **(i)** Extended genomic distances in mechanoCRE interactions. Boxplots showing the distribution of genomic distances between CRE-target gene pairs for distal mechanoCREs (coral) and distal static CREs (grey). *P*-values were calculated using a two-sided Wilcoxon rank-sum test.

**Table S1.** Chip-seq and Hi-C sequencing metrics for LPS-activated BV2 microglia cultured on compliant (1 kPa) and rigid (23kPa) matrices

H3K9ac Chip-seq

| Sample | LPS+ BV2 1kPa  1 | LPS+ BV2 1kPa  2 | LPS+ BV2 1kPa  3 | LPS+ BV2 23kPa  1 | LPS+ BV2 23kPa  2 | LPS+ BV2 23kPa  3 |
| --- | --- | --- | --- | --- | --- | --- |
| Valid reads number | 14542048 | 24106753 | 52855650 | 25351725 | 14702429 | 74025904 |

In situ Hi-C

| Sample | LPS+ BV2 1kPa  1 | LPS+ BV2 1kPa  2 | LPS+ BV2 23kPa  1 | LPS+ BV2 23kPa  2 | LPS+ BV2 1kPa  merged | LPS+ BV2 23kPa merged |
| --- | --- | --- | --- | --- | --- | --- |
| Total reads | 417,000,316 | 405,764,888 | 331,597,728 | 332,029,000 | 822,765,204 | 663,626,728 |
| Long-range (>20kb) cis reads | 219,822,097 | 188,516,682 | 170,029,977 | 174,834,544 | 408,338,779 | 344,864,521 |
| Trans reads | 118,545,700 | 118,644,502 | 79,665,378 | 83,565,958 | 237,190,202 | 163,231,336 |
| Cis/Trans ratio | 64.965 | 61.374 | 68.095 | 67.66 | 63.256 | 67.874 |
| % Long-range intrachromosomal reads | 52.715 | 46.46 | 51.276 | 52.656 | 49.63 | 51.967 |

**Table S2. Full list of motif enrichment analysis results**

(Motif enrichment results of distal upregulated mechanoCREs relative to static CREs)

| **Highlight** | **Motif image** | **-log10(P)** | **Corresponding TF names** |
| --- | --- | --- | --- |
| **NFIL3** |  | 72 | ATF4, CEBPG, HLF, NFIL3, POU5F1 |
| **NF- κB** |  | 55 | HNF4G, HNF4A, RELA, REL |
| **RUNX2** |  | 51 | RUNX2 |
| **HOXA13** |  | 45 | HOXA13, HOXD13, CDX1 |
| **NFAT5** |  | 44 | POU2F3, POU2F2, POU5F1B, POU2F1, POU5F1, POU3F4, NFAT5 |
|  |  | 44 | SOX13, SOX2 |
|  |  | 43 | SPIC |
|  |  | 42 | NR4A1 |
|  |  | 42 | OSR2 |
|  |  | 40 | KLF1, TBX3 |
|  |  | 40 | SOX17 |
|  |  | 40 | SMAD4, HAND1::TCF3 |
|  |  | 39 | STAT1 |
|  |  | 39 | ZNF740, VEZF1, KLF5, KLF4, MAZ, ZNF148 |
|  |  | 36 | GATA3, DPRX |
|  |  | 36 | SOX17, SOX13 |
|  |  | 35 | HOXA13, TBP |
|  |  | 29 | TFAP2C, TFAP2A |
|  |  | 25 | TEAD2, TEAD3 |

(Motif enrichment results of distal downregulated mechanoCREs relative to static CREs)

| **Highlight** | **Motif image** | **-log10(P)** | **Corresponding TF names** |
| --- | --- | --- | --- |
| **KLF4** |  | 121 | KLF4, KLF1, KLF5 |
| **AP-1** |  | 117 | MEIS1, JUN::JUNB, MEIS3, BATF3, BATF, BATF::JUN, FOSL1::JUND |
| **BACH1** |  | 116 | MAFK, BACH1, BACH1::MAFK, NFE2L1 |
| **MXI1** |  | 112 | MXI1 |
| **YY2** |  | 103 | YY2 |
|  |  | 91 | ZNF684 |
|  |  | 86 | ZNF317 |
|  |  | 83 | USF1, USF2, MXI1 |
|  |  | 82 | CREB1, JUN, JUND, ATF2, RUNX3 |
|  |  | 79 | MSANTD3 |
|  |  | 76 | ZBTB32, ZNF341 |
|  |  | 76 | HIC2, RELB, THAP1 |
|  |  | 75 | LBX1 |
|  |  | 74 | ZBTB7C |
|  |  | 70 | HIF1A |
|  |  | 62 | ZNF282 |
|  |  | 61 | MSX1, MSX2, DLX5, DLX4, LHX2, MSX3, DLX3, DLX6, LHX1, DLX2 |
|  |  | 47 | ZNF740, VEZF1, ZNF148, WT1, KLF4, GLIS2 |
|  |  | 36 | HMBOX1 |

(Motif enrichment results of proximal upregulated mechanoCREs relative to static CREs)

| **Highlight** | **Motif image** | **-log10(P)** | **Corresponding TF names** |
| --- | --- | --- | --- |
| **E2F8** |  | 25 | E2F8, E2F7, E2F1, E2F3 |
| **RUNX2** |  | 15 | RUNX2 |
| **ZBTB14** |  | 14 | ZBTB14 |
| **NF- κB** |  | 13 | REL, RELA |
| **ZBTB14** |  | 9 | ZBTB14 |
|  |  | 8 | ZIC1::ZIC2 |

(Motif enrichment results of proximal downregulated mechanoCREs relative to static CREs)

| **Highlight** | **Motif image** | **-log10(P)** | **Corresponding TF names** |
| --- | --- | --- | --- |
| **ZKSCAN1** |  | 87 | ZKSCAN1 |
| **KLF4** |  | 77 | KLF4, KLF5, KLF1, KLF2 |
| **ZNF135** |  | 71 | ZNF135, ZBTB12 |
| **BACH1** |  | 66 | MAFK, BACH1, MAFG, MAFB, BACH1::MAFK, SMAD2::SMAD3 |
| **ZNF740** |  | 64 | ZNF740 |
|  |  | 64 | NR1I3 |
|  |  | 60 | PRDM1 |
|  |  | 59 | ZBTB14 |
|  |  | 59 | ZBTB32 |
|  |  | 58 | PPARG::RXRA, THRB, NR2F1, PPARA::RXRA, NR2F6, PPARD |
|  |  | 57 | TCF7 |
|  |  | 53 | TEAD4, TEAD3, TEAD1, TEAD2 |
|  |  | 51 | RFX7 |
|  |  | 51 | ZSCAN4 |
|  |  | 49 | NR2F2, NR4A1, NR2F1 |
|  |  | 44 | PLAGL1 |
|  |  | 37 | MAFG, MAFF |
|  |  | 36 | CEBPG, DLX5, CEBPB, CEBPE |
|  |  | 31 | ATF1, GMEB1, BZIP_CREB (G-box-like subclass) |

(Motif enrichment results of upregulated distal mechanoCREs relative to downregulated distal mechanoCREs)

| **Highlight** | **Motif image** | **-log10(P)** | **Corresponding TF names** |
| --- | --- | --- | --- |
| **NFIL3** |  | 60 | ATF4, CEBPG, JUN, NFIL3, HLF |
| **ZBTB12** |  | 40 | ZBTB12 |
| **ZNF274** |  | 38 | ZNF274 |
| **RUNX1** |  | 33 | RUNX1, RUNX2 |
| **TBP** |  | 28 | TBP |
|  |  | 25 | SOX18 |
|  |  | 25 | FOXH1 |
|  |  | 20 | FOXQ1, SRY, BARHL2, SOX5, FOXN3, BARHL1, FOXJ2, FOXG1 |
|  |  | 19 | POU2F2, POU2F3, ETV2, ERG, NFATC1 |
|  |  | 14 | SNAI1, TCF3, TCF12, ASCL1, SNAI3, TCF4, ZEB1 |
|  |  | 8 | HINFP |
|  |  | 4 | SMAD5, SMAD3, SMAD4 |
|  |  | 3 | PLAGL1, MZF1, EBF1 |
|  |  | 2 | MXI1 |

(Motif enrichment results of downregulated distal mechanoCREs relative to upregulated distal mechanoCREs)

| **Highlight** | **Motif image** | **-log10(P)** | **Corresponding TF names** |
| --- | --- | --- | --- |
| **BACH1** |  | 194 | NFE2L1, BACH1, BACH1::MAFK, NFE2L2, MAF::NFE2 |
| **ESR1** |  | 105 | ESR1 |
| **TCFL5** |  | 43 | TCFL5 |
| **TEAD2** |  | 38 | TEAD2, USF1, USF2, TEAD4 |
| **ZFX** |  | 34 | ZFX |
|  |  | 33 | ZBED1, ZBTB33 |
|  |  | 26 | PKNOX2, TGIF2, TGIF1 |
|  |  | 24 | CTCFL |
|  |  | 23 | ELF1, ELF3, ETV1, GABPA, EHF |
|  |  | 21 | MXI1, USF2, TFE3, USF1, SREBF1 |
|  |  | 16 | OSR1, OSR2 |
|  |  | 13 | PAX5, PKNOX1, NR2C1, NR1H4, PBX3, NR4A2, NR2C2 |
|  |  | 9 | RELA, TEAD2, TEAD3, ELK3 |
|  |  | 9 | AHR::ARNT, EGR2, ZSCAN29, ZNF354C, KLF12 |

**Table S3.** Information of inhibitors

| **Inhibitor** | Target | Company | Cat# | Work concentration |
| --- | --- | --- | --- | --- |
| Cytochalasin D | Actin polymerization | Tocris Bioscience | 1233 | 1.5μM |
| C646 | Histone acetyltransferase p300/CBP | Tocris Bioscience | 4200 | 20μM |
| Anacordic acid | Histone acetyltransferase | Sigma | A7236 | 50μM |
| Trichostatin A | Class I and II histone deacetylases | Tocris Bioscience | 1406 | 30 nM |

**Table S4.** Information of antibodies

| **Antibody** | Company | Cat# | Concentration |
| --- | --- | --- | --- |
| Iba1 | Abcam | ab178846 | ICC 1:500  IHC 1:200  Multiplex 1:2000 |
| Lamin A/C | Cell signaling | 4777S | ICC 1:100  WB 1:1000 |
| MATF-A | Invitrogen | 25359 | ICC 1:100  WB 1:1000 |
| iNOS | Thermos fisher | PA1-036 | ICC 1:200  WB 1:1000 |
| iNOS | Invitrogen | PA3-030A | Multiplex 1:200 |
| NF-κBp65 | Cell signaling | 8242 | ICC 1:400  WB 1:1000 |
| HDAC3 | Cell signaling | 3949 | ICC 1:100  WB 1:1000 |
| H3ace | Millipore | 06–599 | ICC 1:100  WB 1:500  Multiplex 1:250 |
| GAPDH | Santa Cruz Biotechnology | sc-47724 | WB 1:1000 |
| β-tubulin | Thermo fisher | 32-2600 | WB 1:1000 |
| SRF | Santacruz | sc-25290 | WB 1:1000 |
| Alexa Fluor 488-phalloidin | Thermos fisher | A12379 | ICC 1:200 (1 unit) |

**Table S5.** Primers for gene expression analysis by real-time qPCR

| **Gene** | **Sequence (‘5-3’)** |
| --- | --- |
| ***GAPDH*** | F: AAAGCTGTGGCGTGATGG |
|  | R: TGTCATCATACTTGGCAGGTTTC |
| ***iNOS*** | F: AGAACGTGTTTACCATGAGGC |
|  | R: GTAGGACAATCCACAACTCGC |
| ***TNF-α*** | F: CTACCTTGTTGCCTCCTCTTT |
|  | R: GAGCAGAGGTTCAGTCATGTAG |
| ***IL-6*** | F: CTTCACAAGTCGGAGGCTTAAT |
|  | R: ACTCCAGGTAGCTATGGTACTC |
| ***EP300*** | F: ACTCTCATCTCCGGCCCTCT |
|  | R: CGCCACCATTGGTTAGTCCCA |
| ***CREBBP*** | F: TGCCTTTGCCAGTGAATCGC |
|  | R: GGTCCCATGGGTGCCTGT |
